# Public Concern About Violence, Firearms, and the COVID-19 Pandemic in California

**DOI:** 10.1001/jamanetworkopen.2020.33484

**Published:** 2021-01-04

**Authors:** Nicole Kravitz-Wirtz, Amanda Aubel, Julia Schleimer, Rocco Pallin, Garen Wintemute

**Affiliations:** 1 University of California Firearm Violence Research Center and Violence Prevention Research Program, Department of Emergency Medicine, University of California Davis School of Medicine, Sacramento

## Abstract

**Importance:**

Violence is a significant public health problem that has become entwined with the coronavirus disease 2019 (COVID-19) pandemic.

**Objective:**

To describe individuals’ concerns regarding violence in the context of the pandemic, experiences of pandemic-related unfair treatment, prevalence of and reasons for firearm acquisition, and changes in firearm storage practices due to the pandemic.

**Design, Setting, and Participants:**

This survey study used data from the 2020 California Safety and Well-being Survey, a probability-based internet survey of California adults conducted from July 14 to 27, 2020. Respondents came from the Ipsos KnowledgePanel, an online research panel with members selected using address-based sampling methods. Responses were weighted to be representative of the adult population of California.

**Main Outcomes and Measures:**

Topics included worry about violence for oneself before and during the pandemic; concern about violence for someone else due to a pandemic-related loss; experiences of unfair treatment attributed to the pandemic; firearm and ammunition acquisition due to the pandemic; and changes in firearm storage practices due to the pandemic.

**Results:**

Of 5018 invited panel members, 2870 completed the survey (completion rate, 57%). Among respondents (52.3% [95% CI, 49.5%-55.0%] women; mean [SD] age, 47.9 [16.9] years; 41.9% [95% CI, 39.3%-44.6%] White individuals), self-reported worry about violence for oneself was significantly higher during the pandemic for all violence types except mass shootings, ranging from a 2.8 percentage point increase for robbery (from 65.5% [95% CI, 62.8%-68.0%] to 68.2% [95% CI, 65.6%-70.7%]; *P* = .008) to a 5.6 percentage point increase for stray bullet shootings (from 44.5% [95% CI, 41.7%-47.3%] to 50.0% [47.3%-52.8%]; *P* < .001). The percentage of respondents concerned that someone they know might intentionally harm themselves was 13.1% (95% CI, 11.5%-15.3%). Of those, 7.5% (95% CI, 4.5%-12.2%) said it was because the other person had experienced a pandemic-related loss. An estimated 110 000 individuals (2.4% [95% CI, 1.1%-5.0%] of firearm owners in the state) acquired a firearm due to the pandemic, including 47 000 new owners (43.0% [95% CI, 14.8%-76.6%] of those who had acquired a firearm). Of owners who stored at least 1 firearm in the least secure way, 6.7% (95% CI, 2.7%-15.6%) said they had adopted this unsecure storage practice in response to the pandemic.

**Conclusions and Relevance:**

In this analysis of findings from the 2020 California Safety and Well-being Survey, the COVID-19 pandemic was associated with increases in self-reported worry about violence for oneself and others, increased firearm acquisition, and changes in firearm storage practices. Given the impulsive nature of many types of violence, short-term crisis interventions may be critical for reducing violence-related harm.

## Key Points

**Questions:**

Is the coronavirus disease 2019 (COVID-19) pandemic associated with changes in individuals’ worry about violence happening to themselves or others, the prevalence of and reasons for firearm and ammunition acquisition, and changes in firearm storage practices?

**Findings:**

In this survey study of 2870 adults in California, worry about multiple types of violence for oneself increased during the pandemic. Individuals expressed concern that someone else might physically harm themselves because of pandemic-related losses; there was an increase in firearm acquisition and in unsecure storage practice of loaded firearms in response to the pandemic.

**Meaning:**

The findings of this study suggest that the COVID-19 pandemic and efforts to lessen its spread have compounded the public health burden of violence.

## Teaser

This survey study describes individuals’ concerns regarding violence in the context of the coronavirus disease 2019 (COVID-19) pandemic, experiences of pandemic-related unfair treatment, prevalence of and reasons for firearm acquisition, and changes in firearm storage practices due to the pandemic.

## Introduction

In the United States in 2018, there were nearly 68 000 violence-related deaths.^1^ An additional 3.3 million people reported having been victims of nonfatal violent crime.^2^ Most deaths (57%) and nearly 471 000 nonfatal violent victimizations involved a firearm.^1,2^ Black, Indigenous, Latinx, and other communities of color endure a disproportionate share of this burden.^3,4,5^ It is reasonable to expect that the emergence and progression of the coronavirus disease 2019 (COVID-19) pandemic—with more than 11 million confirmed cases and more than 270 000 deaths nationally as of December 2, 2020^6^—combined with the social, psychological, and economic fallout associated with efforts to lessen its spread, have intensified violence-related harms and inequities therein.

There are multiple possible mechanisms through which the pandemic may be associated with changes in the salience of, or concern about, violence and violence exposure. Pandemic-induced social isolation, hopelessness, and loss, particularly for people with existing mental health conditions, such as depression, may result in thoughts of suicide. Interpersonal violence in the home may increase in frequency and severity as household members, including intimate partners, children, and vulnerable elders, spend more time at home together under high-stress conditions. Having a firearm readily available in these situations creates additional risk.^7,8,9,10^

The pandemic has also worsened long-standing injustices rooted in systemic racism and other oppressive systems of power that contribute to the underlying conditions (eg, poverty, unemployment, and lack of available resources) that elevate risk and compound the consequences of community violence.^11,12,13,14^ Fear and scapegoating associated with COVID-19 may increase bias-motivated unfair treatment.^15,16^ During the summer of 2020, largely peaceful^17^ protests decrying structural inequities, which contribute to both police violence and the uneven burden of COVID-19, have been met, at times, by law enforcement use of crowd-control weapons^18^ and heavily armed white supremacist and far-right vigilantes.^19^

While most major news sources reported initial decreases in violent incidents, as measured by local police calls for service, following pandemic-related lockdowns and stay-at-home orders, the latest indications are that more serious acts of violence, particularly those involving firearms, have remained the same or increased.^20^ In addition to a marked increase in shootings in several large cities across the country,^21^ the pandemic appears to have fueled a surge in firearm background checks, an established proxy for firearm sales. Previous spikes in purchasing, such as those following mass shootings and political elections, have been associated with increased firearm violence.^22^ Similarly, recent research suggests an excess of 2.1 million firearm purchases during the first 3 months of the pandemic, corresponding with an additional 216 to 1335 fatal and nonfatal firearm-related injuries nationwide.^23^

However, the lack of data capturing individuals’ self-reported experiences of, and behaviors associated with, violence in the context of the pandemic has limited our understanding of the intersection of these coinciding public health problems. One of the only past studies to survey individuals who purchased a firearm due to the pandemic relied on a nonrepresentative sample of respondents who did not reflect the sociodemographic profile of most firearm owners.^24^ The current study provides what is, to our knowledge, the first population-representative estimates of individuals’ worry about violence for themselves before and during the pandemic; concern someone they know might harm themselves or others due to a pandemic-related loss; experiences of unfair treatment related to the pandemic; firearm and ammunition purchasing; and changes in firearm storage practices due to the pandemic.

## Methods

Data for this survey study come from the 2020 wave of the California Safety and Well-being Survey, a recurrent statewide survey on firearm ownership and exposure to violence and its consequences in California. The 2020 California Safety and Well-being Survey was designed by the University of California Firearm Violence Research Center and the Violence Prevention Research Program, both at the University of California Davis, and administered online from July 14 to July 27, 2020, by the survey research firm Ipsos.^25^ The 2020 California Safety and Well-being Survey was approved by the University of California Davis institutional review board. Participants read informed consent language, and study initiation constituted consent. This study followed the American Association for Public Opinion Research (AAPOR) reporting guideline.

Respondents were drawn from the Ipsos KnowledgePanel, an online research panel that has been widely used in population-based research, including other studies related to firearm ownership.^26,27^ To establish a nationally representative panel, members are recruited on an ongoing basis through probability-based sampling with random digit dialing and address-based sampling from the US Postal Service’s Delivery Sequence File. A probability proportional to size procedure was used to select a study-specific sample. All members who were aged 18 years and older and residents of California, except those currently serving in the US Armed Forces, were eligible to participate. Invitations were sent by email; automatic reminders were emailed to nonresponders 3 days later. Of 5018 panel members invited to participate, 2870 completed the survey, yielding a 57% completion rate. The median survey completion time was 26 minutes.

A final survey weight variable provided by Ipsos adjusted for the initial probability of selection into KnowledgePanel and for survey-specific nonresponse and overcoverage or undercoverage using poststratification raking ratio adjustments based on cross-classifications of age, sex, race/ethnicity, education, household income, language proficiency, and geographic region within California. Respondents tended to be older, men, and non-Latinx individuals and to have more years of education and higher income than nonresponders (eAppendix 1 in the Supplement). With weighting, the sample is designed to be statistically representative of the noninstitutionalized adult population of California as reflected in the 2018 American Community Survey.

Survey questions for this study covered 4 broad domains, as follows: (1) worry about violence happening to oneself, by type of violence (ie, homicide, suicide, mass shooting, assault, robbery, police violence, accidental shooting, and stray bullet shooting) and incident location, before and during the pandemic; (2) concern that someone else might physically harm another person or themselves in response to a pandemic-related loss; (3) experiences of unfair treatment attributed to the pandemic (eg, being threatened, harassed, or physically assaulted); and (4) firearm and ammunition acquisition and firearm storage practices (among current firearm owners) in response to the pandemic. Detailed survey items and response options are in eAppendix 2 in the Supplement. Item nonresponse for key measures was small (<1.5%). Sociodemographic information was collected as part of ongoing panel membership and merged with individual respondents.

### Statistical Analysis

To generate statewide prevalence estimates, we calculated weighted percentages and 95% CIs for each measure or cross-tabulation of measures using the survey and weighting commands in Stata version 15.1 (StataCorp). Using a retrospective pre-post design, respondents were asked to simultaneously report on their experiences during the pandemic and to reflect on the period before the pandemic. Differences in these measures, overall and by respondent characteristics, were examined using weighted repeated measures multinomial logistic regression models, including interactions between respondent characteristics and an indicator for the period of reference (during vs before the pandemic). The margins command was used to generate prevalence differences and 95% CIs. All statistical tests were 2-tailed, and statistical significance was set at *P* < .05.

## Results

Of 2870 respondents (52.3% [95% CI, 49.5%-55.0%] women; mean [SD] age, 47.9 [16.9] years; 49.1% [95% CI, 39.3%-44.6%] White individuals), 39.8% (95% CI, 37.1%-42.6%) reported that they personally know someone who had tested positive for COVID-19; 1.3% (95% CI, 0.9%-2.0%) reported that they themselves had tested positive, while 4.2% (95% CI, 3.2%-5.3%) reported that they had been sick with COVID-19 but had not been tested. Additional sociodemographic and firearm ownership–related characteristics of respondents appear in eAppendix 3 in the Supplement.

### Worry About Violence

The percentage of respondents who reported that they were somewhat or very worried about violence happening to them was significantly higher during (vs before) the pandemic for all violence types except mass shootings, ranging from a 2.8 percentage point increase for robbery (from 65.5% [95% CI, 62.8%-68.0%] to 68.2% [95% CI, 65.6%-70.7%]; *P* = .008) to 5.4 percentage points each for police violence (from 45.3% [95% CI, 42.5%-48.1%] to 50.6% [95% CI, 47.8%-53.45]; *P* < .001) and unintentional shootings (from 42.7% [95% CI, 39.9%-45.5%] to 48.0% [95% CI, 45.3%-50.9%]; *P* < .001) and 5.6 percentage points for stray bullet shootings (from 44.5% [95% CI, 41.7%-47.3%] to 50.0% [47.3%-52.8%]; *P* < .001) (Table 1). In contrast, worry about mass shootings declined 4.6 percentage points (from 59.9% [95% CI, 57.3%-62.6%] to 55.3% [95% CI, 52.6%-58.1%]; *P* < .001) during the pandemic.

**Table 1.** Prevalence of Worry About Violence, Before and During the Coronavirus Disease 2019 Pandemic, by Violence Type and Location Among 2870 Respondents to the 2020 California Safety and Well-being Survey

Violence	Respondents, % (95% CI)							
	Before the pandemic		During the pandemic					
	Not worried	Somewhat or very worried	Not worried	Somewhat or very worried	Difference among those not worried, percentage points	*P* value	Difference among those somewhat or very worried, percentage points	*P* value
Type								
Homicide	54.7 (51.9-57.5)	44.0 (41.2-46.9)	51.2 (48.4-54.0)	47.6 (44.8-50.4)	–3.5	.001	3.6	.001
Suicide	74.6 (71.9-77.1)	24.5 (22.0-27.2)	71.0 (68.3-73.7)	27.7 (25.1-30.5)	–3.5	.001	3.2	.002
Mass shooting	39.1 (36.5-41.7)	59.9 (57.3-62.6)	43.2 (40.5-46.0)	55.3 (52.6-58.1)	4.2	<.001	–4.6	<.001
Assault	39.9 (37.3-42.6)	59.1 (56.4-61.8)	36.5 (33.9-39.2)	62.4 (59.7-65.1)	–3.4	.001	3.3	.002
Robbery	33.5 (31.0-36.2)	65.5 (62.8-68.0)	30.8 (28.4-33.4)	68.2 (65.6-70.7)	–2.7	.008	2.8	.008
Police violence	53.7 (50.8-56.5)	45.3 (42.5-48.1)	48.1 (45.4-50.9)	50.6 (47.8-53.4)	–5.5	<.001	5.4	<.001
Unintentional shooting	56.2 (53.3-59.0)	42.7 (39.9-45.5)	51.0 (48.2-53.8)	48.0 (45.3-50.9)	–5.2	<.001	5.4	<.001
Stray bullet shooting	54.7 (51.9-57.5)	44.5 (41.7-47.3)	48.8 (46.0-51.6)	50.0 (47.3-52.8)	–5.9	<.001	5.6	<.001
Location								
Home	70.6 (67.9-73.1)	27.9 (25.4-30.5)	69.4 (66.7-72.0)	29.1 (26.5-31.7)	–1.2	.29	1.2	.29
Neighborhood	50.0 (47.3-52.8)	48.8 (46.0-51.6)	46.5 (43.7-49.2)	52.2 (49.4-55.0)	–3.6	.002	3.4	.003
Somewhere else	27.2 (24.8-29.7)	71.3 (68.8-73.7)	26.7 (24.3-29.1)	71.7 (69.2-74.1)	–0.1	.65	0.0	.71

The percentage of respondents who reported being somewhat or very worried about violence happening to them in their neighborhood significantly increased during the pandemic, from 48.8% (95% CI, 46.0%-51.6%) to 52.2% (95% CI, 49.4%-55.0%) (*P* = .003); there was no change in worry about violence in the home or somewhere else (Table 1). Among those who expressed worry about violence in their home, neighborhood, or somewhere else before and/or during the pandemic, similar percentages reported that their worry was at least in part due to their spouse or intimate partner (9.7% [95% CI, 7.9%-11.9%] before and 9.0% [95% CI, 7.3%-11.1%] during) (eAppendix 4 in the Supplement). Of those, most were somewhat or very willing to ask for help from a domestic violence hotline (75.4%; 95% CI, 65.5%-83.3%), family member or friend (86.8%; 95% CI, 77.1%-92.8%), and law enforcement (89.5%; 95% CI, 80.5%-94.6%) (eAppendix 5, eAppendix 6, and eAppendix 7 in the Supplement). The share of respondents who reported worry about violence by sociodemographic characteristics and firearm ownership status appears in eAppendices 4 to 18 in the Supplement.

### Concern About Violence for Others

Of the 12.1% (95% CI, 10.4%-14.1%) of respondents who reported concern that someone they know might physically hurt another person on purpose, 1.8% (95% CI, 0.7%-4.4%) reported that their concern was at least in part because the person had experienced a major loss (eg, loss of someone they cared about, a job, or housing) related to the pandemic (Table 2). Likewise, of the 13.3% (95% CI, 11.5%-15.3%) of respondents who reported concern that someone they know might physically hurt themselves on purpose, 7.5% (95% CI, 4.5%-12.2%) reported that their concern was at least in part because the person had experienced a pandemic-related loss.

**Table 2.** Prevalence of Concern That Someone They Know Might Physically Hurt Another Person or Themselves on Purpose, Reasons for Concern, and Firearm Access Among 2870 Respondents to the 2020 California Safety and Well-being Survey

Factor	Respondents, % (95% CI)	
	Concern that someone they know might hurt another person	Concern that someone they know might hurt themselves
Total	12.1 (10.4-14.1)	13.3 (11.5-15.3)
Reasons for concern		
Other non–COVID-19 reasons only	98.2 (95.6-99.3)	92.5 (87.8-95.5)
COVID-19–related loss	1.8 (0.7-4.4)	7.5 (4.5-12.2)
Firearm access		
Yes	0	6.0 (1.2-25.8)
No	10.2 (1.8-41.1)	36.6 (17.2-61.6)
Don’t know	89.8 (58.9-98.2)	57.4 (32.9-78.7)

Abbreviation: COVID-19, coronavirus disease 2019.

Among respondents whose concerns were due to a pandemic-related loss, most said they did not know whether the other person had access to a firearm (other-directed harm: 89.8% [95% CI, 58.9%-98.2%]; self-directed harm: 57.4% [95% CI, 32.9%-78.7%]). Of respondents who were concerned that someone they know might harm themselves due to a pandemic-related loss, 6.0% (95% CI, 1.2%-25.8%) said the person had access to a firearm (Table 2).

### Experiences of Unfair Treatment

The percentage of respondents who reported that they had experienced at least 1 form of unfair treatment in the past 12 months was 69.2% (95% CI, 66.6%-71.7%) (Table 3). Of those, 7.4% (95% CI, 5.6%-9.6%) said the unfair treatment was related to the pandemic. Asian respondents most often reported pandemic-related unfair treatment: 17.2% (95% CI, 10.2%-27.5%) of Asian respondents who experienced unfair treatment said it was related to the pandemic, compared with 10.7% (95% CI, 2.2%-39.5%) of those who identified as multiracial or other race, 7.5% (95% CI, 2.4%-20.5%) of Black respondents, 7.4% (95% CI, 5.1%-10.7%) of White respondents, and 3.0% (95% CI, 1.5%-5.9%) of Latinx respondents.

**Table 3.** Prevalence of Unfair Treatment in the Past 12 Months, Overall and Due to the COVID-19 Pandemic, by Race/Ethnicity, Among 2870 Respondents to the 2020 California Safety and Well-being Survey

Race/ethnicity^a^	Respondents, % (95% CI)	
	Experienced unfair treatment	Experienced COVID-19–related unfair treatment
Total	69.2 (66.6-71.7)	7.4 (5.6-9.6)
Asian, non-Latinx	66.9 (58.4-74.4)	17.2 (10.2-27.5)
Black, non-Latinx	75.9 (63.8-85.0)	7.5 (2.4-20.5)
Latinx	69.1 (64.3-73.5)	3.0 (1.5-5.9)
Multiracial or other, non Latinx	69.2 (50.7-83.1)	10.7 (2.2-39.5)
White, non-Latinx	69.1 (65.7-72.3)	7.4 (5.1-10.7)

Abbreviation: COVID-19, coronavirus disease 2019.

^a^ Respondents were asked, “Are you Spanish, Hispanic, or Latino?” and then, “Please indicate what you consider your race to be. We appreciate your effort to describe your background using these US Census Bureau categories.” Response options were White, Black or African American, American Indian or Alaska Native, Asian, Native Hawaiian or other Pacific Islander, and some other race. Respondents could select all answers that applied. American Indian or Alaska Native, Native Hawaiian or other Pacific Islander, and some other race were combined by Ipsos into the category other.

### Firearm Acquisition and Storage Practices

The percentage of respondents who reported that they or someone else in their household owned firearms was 23.5% (95% CI, 21.3%-25.9%); 15.2% (95% CI, 13.4%-17.2%) of respondents reported that they were a firearm owner (eAppendix 3 in the Supplement). Among owners, 2.4% (95% CI, 1.1%-5.0%) reported that they had acquired a firearm in response to the pandemic, while 8.5% (95% CI, 5.0%-14.0%) of owners, including all of those who had acquired a firearm, said that they had purchased ammunition in response to the pandemic (Table 4). Among those who had acquired a firearm in response to the pandemic, 43.0% (95% CI, 14.8%-76.6%) reported that they did not already own a firearm. Extrapolating to the population of adults in California (30.1 million in 2018), we estimated that approximately 110 000 Californians acquired firearms in response to the pandemic, including 47 000 new owners.

**Table 4.** Prevalence of Firearm and Ammunition Acquisition in Response to the Coronavirus Disease 2019 Pandemic and Related Characteristics Among 529 Firearm Owners in the 2020 California Safety and Well-being Survey

Behavior	Respondents, % (95% CI)	
	Firearms	Ammunition
Acquired in response to the pandemic	2.4 (1.1-5.0)	8.5 (5.0-14.0)
Did not already own a firearm	43.0 (14.8-76.6)	NA
Reasons for acquisition^a^		
Lawlessness	75.9 (27.6-96.3)	77.1 (46.6-92.8)
Prisoner releases	56.1 (22.0-85.3)	49.2 (24.6-74.2)
Government going too far	49.2 (17.7-81.3)	38.5 (17.8-64.4)
Government collapse	38.0 (12.2-73.0)	52.9 (27.6-76.7)
Gun stores closing	31.1 (9.7-65.4)	35.8 (15.3-63.3)
Hunting	10.5 (1.4-49.4)	7.2 (1.8-24.6)
Sport shooting	10.5 (1.4-49.4)	8.1 (2.7-22.2)
Some other reason	0	4.0 (0.9-15.9)
Firearm storage		
All guns unloaded and locked up	62.7 (56.2-68.7)	NA
Change in response to pandemic	0	NA
≥1 gun(s) loaded and not locked up	18.0 (13.1-24.1)	NA
Change in response to the pandemic	6.7 (2.7-15.6)	NA
Some other way	18.6 (14.8-23.1)	NA

Abbreviation: NA, not applicable.

^a^ Reasons for acquisition are not mutually exclusive.

The most common reason given for firearm acquisition in response to the pandemic was worry about lawlessness (75.9%; 95% CI, 27.6%-96.3%), followed by worry about prisoner releases (56.1%; 95% CI, 22.0%-85.3%), the government going too far (49.2%; 95% CI, 17.7%-81.3%), government collapse (38.0%; 95% CI, 12.2%-73.0%), and gun stores closing (31.1%; 95% CI, 9.7%-65.4%) (Table 4). Reasons for ammunition purchases in response to the pandemic were similar. Firearm owners (vs nonowners) and those who had acquired a firearm in response to the pandemic (vs nonowners and owners without a pandemic-related acquisition) also had the largest percentage increases in their level of worry about multiple types of violence during (vs before) the pandemic (eAppendices 8-18 in the Supplement).

Among firearm owners, 62.7% (95% CI, 56.2%-68.7%) reported that they currently store all of their firearms in the most secure way (ie, unloaded and locked up), 18.0% (95% CI, 13.1%-24.1%) that they store at least 1 firearm in the least secure way (ie, loaded and not locked up), and the remainder (18.6%; 95% CI, 14.8%-23.1%) that they store their firearms in some other way (eg, unloaded but not locked up) (Table 4). Of owners who currently store at least 1 firearm in the least secure way, 6.7% (95% CI, 2.7%-15.6%) reported that this reflected a change in storage practices because of the pandemic. Of those, approximately half (53.0%; 95% CI, 17.2%-86.0%) lived in households with children or teenagers.

## Discussion

Violence is a significant public health problem that touches the lives of far more people than is typically recognized. Violence affects people not only through direct involvement but also through indirect and vicarious experiences that may increase the salience of, and concern about, violence across networks of individuals, families, and entire communities. This state-representative survey of California adults examined self-reported concerns about, experiences of, and behaviors associated with violence in the context of the COVID-19 pandemic. Our findings add support to a growing body of research suggesting that the pandemic, and efforts to lessen its spread, have compounded the burden of violence-related harms.

Our respondents expressed higher levels of worry about violence during (vs before) the pandemic, ranging from 2.8 to 5.6 percentage point increases in the estimated statewide prevalence of adults who reported that they were somewhat or very worried about multiple types of interpersonal violence (ie, robbery, assault, homicide, police violence), suicide, and unintentional firearm injury happening to them. As expected,^15,16^ self-reported experiences of unfair treatment attributed to the pandemic were also reported and disproportionately common among Asian respondents. In addition to worry about violence for oneself, 13.3% of respondents reported concern that someone they know might physically harm themselves on purpose. Of those, 7.5% said this concern was at least in part because the other person had experienced a pandemic-related loss.

The processes underlying changes in worry about violence for oneself and others during the pandemic—as well as the absence of an observed change in worry about violence due to an intimate partner—warrant further investigation. Past research suggests that neighborhood social cohesion and collective efficacy inform both perceptions of safety and actual levels of crime and violence^28^; however, pandemic-induced social distancing and stay-at-home orders, combined with more general fears regarding COVID-19 danger and contamination,^29^ may make such social bonds and informal social controls difficult to establish and maintain. In prior work, worry about violence has been associated with higher perceived stress and higher depressive symptoms^30^ as well as with health-risk behaviors, such as firearm carrying, that may increase the risk of future violence involvement.^31^

Mounting concern about violence may also stem from the surge in firearm background checks, a proxy for firearm sales, in the months coinciding with the pandemic.^23^ To our knowledge, however, this was the first population-representative study to estimate the prevalence of and motivations for ammunition and firearm acquisition in direct response to the pandemic. We found that 8.5% of firearm owners in California purchased ammunition in response to the pandemic, including an estimated 110 000 individuals who also acquired firearms (2.4% of owners in the state). Of those, an estimated 47 000 were new owners, who may have little past experience or training with firearms. Consistent with the most common reasons for firearm ownership generally,^24,26,32^ respondents who acquired firearms due to the pandemic usually did so for self-protection: three-quarters (75.9%) indicated worry about lawlessness and more than half (56.1%) endorsed worry about prisoner releases.

Although respondents’ perceived need for self-protection continued to motivate firearm ownership amid the pandemic, an extensive body of evidence suggests that the presence of a firearm in the home elevates risk for firearm-related harm, particularly unintentional shootings (often involving children or teenagers), intimate homicide against women, and completed suicide.^7,8,9,10^ Previous spikes in firearm purchasing have been associated with increased firearm violence,^22^ and recent evidence suggests a similar association exists during the pandemic, particularly for suicide.^23,33^ In addition, past research suggests that people who own firearms primarily for protection are more likely to store firearms in the home loaded and/or not locked up,^34^ an independent risk factor for firearm injury and death. Our findings suggest the pandemic may be associated with increases in this risk: an estimated 55 000 people (1.2% of owners in the state) who stored at least 1 firearm loaded and not locked up reported adopting this unsecure storage practice in response to the pandemic.

Taken together, our findings add support to comprehensive public health–oriented prevention and intervention strategies designed to address the enduring psychological trauma associated with exposure to and worry about violence as well as the intermediary (eg, firearm ownership and storage) and upstream (eg, socioeconomic characteristics, education, and the environment) factors associated with violence risk. More immediately, given the impulsive nature of many types of violence and the multiple acute disruptions associated with the pandemic, short-term crisis interventions, such as options for temporary firearm storage outside the home, extreme risk protection orders, and efforts involving violence intervention workers in hospitals and communities, may be particularly critical for reducing the burden of violence.

### Limitations

This study has some limitations. First, we relied on self-reported data, which is subject to social desirability, nonresponse, and recall biases. However, several administrative data sources provide an opportunity to broadly assess the validity of our estimates. The Johns Hopkins Coronavirus Resource Center documented 346 000 to 458 000 confirmed cases of coronavirus in California at the time our survey was in the field, close to our estimate of 391 000 after extrapolating to the population of adults in the state. Similarly, National Instant Criminal Background Check System data show approximately 557 000 people underwent a firearm background check in California from March through June 2020 compared with 465 000 during the same period in 2019. This amounts to a year-over-year increase of 92 000, close to our estimate of 110 000 pandemic-related firearm acquisitions.

Second, given state-level differences in infection rates and in efforts to lessen the spread and impacts of the COVID-19 pandemic as well as California’s relatively low rates of firearm ownership and more comprehensive firearm regulations, our findings might not be generalizable to other states. However, COVID-19 is a near ubiquitous exposure across the United States, and nationally representative studies have similarly found deleterious associations of the pandemic with psychological health^29,35^ as well as nationwide increases in firearm purchasing during the pandemic.^23^

Third, we used a retrospective pre-post approach to compare responses before and during the pandemic, which may inaccurately reflect the associations with the pandemic if respondents’ knowledge or experiences of the pandemic led them to interpret questions in a qualitatively different manner at survey administration than they would have in the period before the pandemic. However, some research suggests that when individuals are asked to respond to questions about a particular subject after they have some basic knowledge of or experience with the subject itself, they are better able to accurately reflect on the degree of change.^36^

## Conclusions

This state-representative survey study assessed the near-term associations of the pandemic on individual perceptions, motivations, and behaviors related to violence and firearm ownership in California. It found that the COVID-19 pandemic was associated with increases in self-reported worry about violence for oneself and others, increased firearm acquisition, and changes in firearm storage practices. These findings can inform prevention and intervention efforts now and following other societal shocks that exacerbate persistent structural, economic, and social inequities that are associated with violence and its consequences as well as lay the groundwork for more comprehensive research and prevention efforts in the future.

## Notes

Supplement. **eAppendix 1. **Characteristics of 5018 Respondents and Nonrespondents, 2020 California Safety and Well-being Survey **eAppendix 2. **Survey Items and Response Options, 2020 California Safety and Well-being Survey **eAppendix 3. **Characteristics of 2870 Respondents, 2020 California Safety and Well-being Survey **eAppendix 4. **Percentage of Respondents Worried About Intimate Partner Violence Happening to Them Before and During the COVID-19 Pandemic, Among Those Worried About a Violent Event Happening to Them, 2020 California Safety and Well-being Survey **eAppendix 5. **Percentage of Respondents Willing to Ask for Help From a Hotline, Among Those Worried About Intimate Partner Violence Happening to Them, 2020 California Safety and Well-being Survey **eAppendix 6. **Percentage of Respondents Willing to Ask for Help From Family, Among Those Worried About Intimate Partner Violence Happening to Them, 2020 California Safety and Well-being Survey **eAppendix 7. **Percentage of Respondents Willing to Ask for Help From Law Enforcement, Among Those Worried About Intimate Partner Violence Happening to Them, 2020 California Safety and Well-being Survey **eAppendix 8. **Percentage of Respondents Worried About Homicide Happening to Them Before and During the COVID-19 Pandemic, 2020 California Safety and Well-being Survey **eAppendix 9. **Percentage of Respondents Worried About Suicide Happening to Them Before and During the COVID-19 Pandemic, 2020 California Safety and Well-being Survey **eAppendix 10. **Percentage of Respondents Worried About a Mass Shooting Happening to Them Before and During the COVID-19 Pandemic, 2020 California Safety and Well-being Survey **eAppendix 11.** Percentage of Respondents Worried About Assault Happening to Them Before and During the COVID-19 Pandemic, 2020 California Safety and Well-being Survey **eAppendix 12. **Percentage of Respondents Worried About Robbery Happening to Them Before and During the COVID-19 Pandemic, 2020 California Safety and Well-being Survey **eAppendix 13. **Percentage of Respondents Worried About Police Violence Happening to Them Before and During the COVID-19 Pandemic, 2020 California Safety and Well-being Survey **eAppendix 14. **Percentage of Respondents Worried About an Unintentional Shooting Happening to Them Before and During the COVID-19 Pandemic, 2020 California Safety and Well-being Survey **eAppendix 15. **Percentage of Respondents Worried About Being Hit by a Stray Bullet Before and During the COVID-19 Pandemic, 2020 California Safety and Well-being Survey **eAppendix 16. **Percentage of Respondents Worried About a Violent Event Happening to Them in Their Home Before and During the COVID-19 Pandemic, 2020 California Safety and Well-being Survey **eAppendix 17. **Percentage of Respondents Worried About a Violent Event Happening to Them in Their Neighborhood Before and During the COVID-19 Pandemic, 2020 California Safety and Well-being Survey **eAppendix 18. **Percentage of Respondents Worried About a Violent Event Happening to Them Somewhere Else Before and During the COVID-19 Pandemic, 2020 California Safety and Well-being Survey
