# Supplementary material for: Public Concern About Violence, Firearms, and the COVID-19 Pandemic in California
Source: JAMA Netw Open. 2021 Jan 4;4(1):e2033484. doi: 10.1001/jamanetworkopen.2020.33484 (PMC7783542; doi:10.1001/jamanetworkopen.2020.33484)
Supplement: Supplement. — eAppendix 1. Characteristics of 5018 Respondents and Nonrespondents, 2020 California Safety and Well-being Survey eAppendix 2. Survey Items and Response Options, 2020 California Safety and Well-being Survey eAppendix 3. Characteristics of 2870 Respondents, 2020 California Safety and Well-being Survey eAppendix 4. Percentage of Respondents Worried About Intimate Partner Violence Happening to Them Before and During the COVID-19 Pandemic, Among Those Worried About a Violent Event Happening to Them, 2020 California Safety and Well-being Survey eAppendix 5. Percentage of Respondents Willing to Ask for Help From a Hotline, Among Those Worried About Intimate Partner Violence Happening to Them, 2020 California Safety and Well-being Survey eAppendix 6. Percentage of Respondents Willing to Ask for Help From Family, Among Those Worried About Intimate Partner Violence Happening to Them, 2020 California Safety and Well-being Survey eAppendix 7. Percentage of Respondents Willing to Ask for Help From Law Enforcement, Among Those Worried About Intimate Partner Violence Happening to Them, 2020 California Safety and Well-being Survey eAppendix 8. Percentage of Respondents Worried About Homicide Happening to Them Before and During the COVID-19 Pandemic, 2020 California Safety and Well-being Survey eAppendix 9. Percentage of Respondents Worried About Suicide Happening to Them Before and During the COVID-19 Pandemic, 2020 California Safety and Well-being Survey eAppendix 10. Percentage of Respondents Worried About a Mass Shooting Happening to Them Before and During the COVID-19 Pandemic, 2020 California Safety and Well-being Survey eAppendix 11. Percentage of Respondents Worried About Assault Happening to Them Before and During the COVID-19 Pandemic, 2020 California Safety and Well-being Survey eAppendix 12. Percentage of Respondents Worried About Robbery Happening to Them Before and During the COVID-19 Pandemic, 2020 California Safety and Well-being Survey eAppendix 13. Percentage of R [file jamanetwopen-e2033484-s001.pdf]

Supplemental Online Content

Kravitz-Wirtz N, Aubel A, Schleimer J, Pallin R, Wintemute G. Public concern about violence, firearms, and the COVID-19 pandemic in California. *JAMA Netw Open*. 2021;4(1):e2033484. doi:10.1001/jamanetworkopen.2020.33484

eAppendix 1. Characteristics of 5018 Respondents and Nonrespondents, 2020 California Safety and Well-being Survey
eAppendix 2. Survey Items and Response Options, 2020 California Safety and Well-being Survey
eAppendix 3. Characteristics of 2870 Respondents, 2020 California Safety and Well-being Survey
eAppendix 4. Percentage of Respondents Worried About Intimate Partner Violence Happening to Them Before and During the COVID-19 Pandemic, Among Those Worried About a Violent Event Happening to Them, 2020 California Safety and Well-being Survey
eAppendix 5. Percentage of Respondents Willing to Ask for Help From a Hotline, Among Those Worried About Intimate Partner Violence Happening to Them, 2020 California Safety and Well-being Survey
eAppendix 6. Percentage of Respondents Willing to Ask for Help From Family, Among Those Worried About Intimate Partner Violence Happening to Them, 2020 California Safety and Well-being Survey
eAppendix 7. Percentage of Respondents Willing to Ask for Help From Law Enforcement, Among Those Worried About Intimate Partner Violence Happening to Them, 2020 California Safety and Well-being Survey
eAppendix 8. Percentage of Respondents Worried About Homicide Happening to Them Before and During the COVID-19 Pandemic, 2020 California Safety and Well-being Survey
eAppendix 9. Percentage of Respondents Worried About Suicide Happening to Them Before and During the COVID-19 Pandemic, 2020 California Safety and Well-being Survey
eAppendix 10. Percentage of Respondents Worried About a Mass Shooting Happening to Them Before and During the COVID-19 Pandemic, 2020 California Safety and Well-being Survey
eAppendix 11. Percentage of Respondents Worried About Assault Happening to Them Before and During the COVID-19 Pandemic, 2020 California Safety and Well-being Survey
eAppendix 12. Percentage of Respondents Worried About Robbery Happening to Them Before and During the COVID-19 Pandemic, 2020 California Safety and Well-being Survey

© 2021 Kravitz-Wirtz N et al. *JAMA Network Open.*

eAppendix 13. Percentage of Respondents Worried About Police Violence Happening to Them Before and During the COVID-19 Pandemic, 2020 California Safety and Well-being Survey
eAppendix 14. Percentage of Respondents Worried About an Unintentional Shooting Happening to Them Before and During the COVID-19 Pandemic, 2020 California Safety and Well-being Survey
eAppendix 15. Percentage of Respondents Worried About Being Hit by a Stray Bullet Before and During the COVID-19 Pandemic, 2020 California Safety and Well-being Survey
eAppendix 16. Percentage of Respondents Worried About a Violent Event Happening to Them in Their Home Before and During the COVID-19 Pandemic, 2020 California Safety and Well-being Survey
eAppendix 17. Percentage of Respondents Worried About a Violent Event Happening to Them in Their Neighborhood Before and During the COVID-19 Pandemic, 2020 California Safety and Well-being Survey
eAppendix 18. Percentage of Respondents Worried About a Violent Event Happening to Them Somewhere Else Before and During the COVID-19 Pandemic, 2020 California Safety and Well-being Survey

This supplemental material has been provided by the authors to give readers additional information about their work.

© 2021 Kravitz-Wirtz N et al. *JAMA Network Open.*

eAppendix 1. Characteristics of 5018 Respondents and Nonrespondents, 2020 California Safety and Well-being Survey

	Respondents (n=2870)				Non-respondents (n=2148)	
	Unweighted n	Unweighted %	Weighted % [95% CI]		n	%
Age						
18-29	200	7.0	16.3 [14.0-18.9]		496	23.1
30-44	511	17.8	29.7 [27.0-32.5]		586	27.3
45-59	738	25.7	26.7 [24.4-29.2]		604	28.1
60+	1421	49.5	27.3 [25.3-29.4]		462	21.5
Race/ethnicity*						
White, Non-Latinx	1615	56.3	41.9 [39.3-44.6]		715	33.3
Black, Non-Latinx	127	4.4	5.8 [4.6-7.3]		107	5.0
Other, Non-Latinx	220	7.7	15.5 [13.4-18.0]		140	6.5
2+ races, Non-Latinx	59	2.1	2.1 [1.4-3.1]		38	1.8
Latinx	849	29.6	34.7 [32.0-37.4]		1148	53.5
Gender						
Male	1506	52.5	47.7 [45.0-50.5]		925	43.1
Female	1364	47.5	52.3 [49.5-55.0]		1223	56.9
Education						
Less than high school	158	5.5	15.4 [13.1-18.0]		234	10.9
High school	364	12.7	20.4 [18.1-23.0]		377	17.6
Some college	938	32.7	32.7 [30.2-35.2]		755	35.2
Bachelor's degree or higher	1410	49.1	31.5 [29.1-33.9]		782	36.4
Income						
Less than $25,000	431	15.0	15.1 [13.2-17.2]		502	23.4
$25,000-$59,999	744	25.9	23.9 [21.7-26.3]		500	23.3
$60,000-$99,999	757	26.4	22.6 [20.4-24.9]		650	30.3
$100,000 or more	938	32.7	38.5 [35.8-41.3]		496	23.1

*Respondents were asked, “Are you Spanish, Hispanic, or Latino?” and then, “Please indicate what you consider your race to be. We appreciate your effort to describe your background using these US Census Bureau categories.” Response options were White, Black or African American, American Indian or Alaska Native, Asian, Native Hawaiian or other Pacific Islander, and Some other race. Respondents could select all answers that apply. American Indian or Alaska Native, Native Hawaiian or other Pacific Islander, and Some other race were combined by Ipsos into an Other category.

© 2021 Kravitz-Wirtz N et al. *JAMA Network Open.*

eAppendix 2. Survey Items and Response Options, 2020 California Safety and Well-being Survey

Q3. **[Accordion Grid]**
In general, **before** the coronavirus epidemic, how worried were you about each of the following violent events happening to **you**?

Scripter: Randomize order of statements. Record order in which statements were shown.

Statements:
1. Homicide
2. Suicide
3. Mass shooting
4. Assault
5. Robbery
6. Police violence
7. Accidental shooting
8. Getting hit by a stray bullet

Responses:
1. Not worried
2. Somewhat worried
3. Very worried 17

Q4. **[Accordion Grid]**
These days, **during** the coronavirus epidemic, how worried are you about each of the following violent events happening to **you**?

Scripter: Same order of items as Q3

Statements:
1. Homicide
2. Suicide
3. Mass shooting
4. Assault
5. Robbery
6. Police violence
7. Accidental shooting
8. Getting hit by a stray bullet

Responses:
1. Not worried
2. Somewhat worried
3. Very worried

Q7. **[Grid, SP Across]**
In general, **before** the coronavirus epidemic, how worried were you about a violent event happening to **you** in each of the following places?

Statements per row:
1. In my home
2. Not in my home but in my neighborhood
3. Somewhere else

Answers in columns:
1. Not worried
2. Somewhat worried
3. Very worried

Q8. **[S]**
You said you were somewhat or very worried about a violent event happening to **you**. Was this worry at least in part because of your spouse or intimate partner?

1. Yes
2. No

© 2021 Kravitz-Wirtz N et al. *JAMA Network Open.*

Q9. **[Grid, SP Across]**
These days, **during** the coronavirus epidemic, how worried are you about a violent event happening to **you** in each of the following places?

Statements per row:
1. In my home
2. Not in my home but in my neighborhood
3. Somewhere else

Answers in columns:
1. Not worried
2. Somewhat worried
3. Very worried

Q10. **[S]**
You said you are somewhat or very worried about a violent event happening to **you**. Is this worry at least in part because of your spouse or intimate partner?

1. Yes
2. No

Q12. **[Accordion Grid]**
How willing are you to ask for help from the following people?

Statements:
1. A domestic violence hotline
2. A family member or trusted friend
3. Law enforcement

Responses:
1. Not at all willing
2. Somewhat willing
3. Very willing

Q35. **[S]** [PROMPT ONCE IF NO RESPONSE GIVEN]
Are you concerned that anyone you know might physically hurt **another person** on purpose? Consider only people you know personally, not people you've only heard about from others or seen in the media.

1. Yes
2. No

Q47. **[M]**
Do any of these things make you concerned this person might physically hurt another person on purpose? Select all that apply.

1. They have suffered a major loss, such as loss of someone they cared about, loss of a job, or loss of housing
2. They have hurt another person before
3. They have hurt themselves before
4. They have trouble with alcohol or drug use
6. They have posted about violence or made threats to hurt another person on social media
7. They have talked about violence or made threats to hurt another person
10. They have damaged property
11. Other, please specify: [Text box]
12. None of the above [E]

Q48. **[S]**
You said they have suffered a major loss. Was this loss related to the coronavirus epidemic or something else?

1. The coronavirus epidemic
2. Something else
3. Both
4. Don’t know

Q52. **[S]**
Does this person have access to a gun?

1. Yes
2. No
3. Don’t know

Q63. **[S]** [PROMPT ONCE IF NO RESPONSE GIVEN]
Are you concerned that anyone you know might physically hurt **themselves** on purpose? Consider only people you know personally, not people you've only heard about from others or seen in the media.

1. Yes
2. No

Q75. **[M]**
Do any of these things make you concerned this person might physically hurt themselves on purpose? Select all that apply.

1. They have suffered a major loss, such as loss of someone they cared about, loss of a job, or loss of housing
2. They have hurt another person before
3. They have hurt themselves before
4. They have trouble with alcohol use or drug use
6. They have posted about violence or made threats to hurt themselves on social media
7. They have talked about violence or made threats to hurt themselves
10. They have damaged property
11. Other, please specify:[Text box] [A]
12. None of the above [A,E]

Q76. **[S]**
You said they have suffered a major loss. Was this loss related to the coronavirus epidemic, or something else?

1. The coronavirus epidemic
2. Something else
3. Both
4. Don’t know

Q78. **[S]**
Does this person have access to a gun?

1. Yes
2. No
3. Don’t know

Q129. **[Accordion Grid]**
In the past 12 months, how often have any of the following things happened to you?

Statements per row:
1. You were treated with less respect than other people
2. You were treated unfairly at restaurants or stores or other public places
3. People criticized your accent or the way you speak
4. People acted as if they think you are not smart
5. People acted as if they are afraid of you
6. People acted as if they think you are dishonest
7. People acted as if they think they are better than you are
8. You were threatened or harassed
9. You were physically assaulted

Answers in columns:
1. Never
2. Rarely
3. Sometimes
4. Often
5. Don’t know

Q132. **[S]** [PROMPT ONCE IF NO RESPONSE GIVEN]

© 2021 Kravitz-Wirtz N et al. *JAMA Network Open.*

Was any of the unfair treatment related to the coronavirus epidemic?

1. Yes
2. No
3. Don't know

Q110. **[S]** [PROMPT ONCE IF NO RESPONSE GIVEN]
Have you acquired any guns in response to the coronavirus epidemic?

1. Yes
2. No

Q111. **[M]**
You said you acquired a gun in response to the coronavirus epidemic. Did you get this gun for any of the specific reasons below? Select all that apply.

1. I'm worried about lawlessness
2. I'm worried about government collapse
3. I'm worried about government going too far
4. I'm worried about prisoner releases
5. I'm worried about getting enough food and need to hunt
6. I have more time on my hands for sport shooting
7. I'm worried about gun stores being closed
8. Other, please specify ___________ [A] [SCRIPTER: PROMPT ONCE IF SELECTED AND NO RESPONSE GIVEN]

Q112. **[S]**
You said you acquired a gun in response to the coronavirus epidemic. Did you already personally own a gun at that time?

1. Yes
2. No

Q113. **[S]** [PROMPT ONCE IF NO RESPONSE GIVEN]
Have you bought ammunition in response to the coronavirus epidemic?

1. Yes
2. No 45

Q114. **[M]**
You said you bought ammunition in response to the coronavirus epidemic. Did you buy this ammunition for any of the specific reasons below? Select all that apply.

1. I'm worried about lawlessness
2. I'm worried about government collapse
3. I'm worried about government going too far
4. I'm worried about prisoner releases
5. I'm worried about getting enough food and need to hunt
6. I have more time on my hands for sport shooting
7. I'm worried about gun stores being closed
8. Other, please specify ___________ [A] [SCRIPTER: PROMPT ONCE IF SELECTED AND NO RESPONSE GIVEN]

Q115. **[M]**
How do you currently store your gun(s)? Select all that apply.

1. Unloaded and locked up (for example, with a trigger lock, cable lock, in a lock box or safe)
2. Loaded and not locked up
3. Some other way

Q116. **[S]**
You said you stored gun(s) unloaded and locked up. Have you always stored gun(s) this way?

1. Yes
2. No

Q117. **[S]**

Is this a change because of the coronavirus epidemic?

1. Yes
2. No

Q118. **[S]**
You said you stored gun(s) loaded and not locked up. Have you always stored gun(s) this way?

1. Yes
2. No

Q119. **[S]**
Is this a change because of the coronavirus epidemic?

1. Yes
2. No

eAppendix 3. Characteristics of 2870 Respondents, 2020 California Safety and Well-being Survey

	n	% [95% CI]
Age		
18-29	200	16.3 [14.0-18.9]
30-44	511	29.7 [27.0-32.5]
45-59	738	26.7 [24.4-29.2]
60+	1421	27.3 [25.3-29.4]
Race/ethnicity*		
White, Non-Latinx	1615	41.9 [39.3-44.6]
Black, Non-Latinx	127	5.8 [4.6-7.3]
Asian, Non-Latinx	208	14.4 [12.3-16.8]
Other & 2+ races, Non-Latinx	71	3.2 [2.3-4.5]
Latinx	849	34.7 [32.0-37.4]
Gender		
Male	1506	47.7 [45.0-50.5]
Female	1364	52.3 [49.5-55.0]
Education		
Less than high school	158	15.4 [13.1-18.0]
High school	364	20.4 [18.1-23.0]
Some college	938	32.7 [30.2-35.2]
Bachelor's degree	775	17.5 [15.7-19.4]
Advanced degree	635	14.0 [12.4-15.8]
Income		
Less than $25,000	431	15.1 [13.2-17.2]
$25,000-$59,999	744	23.9 [21.7-26.3]
$60,000-$99,999	757	22.6 [20.4-24.9]
$100,000 or more	938	38.5 [35.8-41.3]
Household gun ownership		
No guns in home	1989	71.4 [68.8-73.8]
Gun owner	529	15.2 [13.4-17.2]
Non-owner living with owner	219	8.3 [6.9-10.0]
Acquired firearms due to the coronavirus pandemic		
Non-gun owner	2341	84.8 [82.8-86.6]
Gun owner, no purchase	519	14.8 [13.0-16.8]
Gun owner, purchase	10	0.4 [0.2-0.8]
Tested positive for or been sick with coronavirus		
Yes	46	1.3 [0.9-2.0]
No	2689	94.0 [92.7-95.1]
I was sick but didn't get tested	124	4.2 [3.2-5.3]
Number of people that you know who have tested positive for coronavirus		
0	1639	54.0 [51.2-56.8]
1-2	696	24.0 [21.7-26.4]
3-5	296	10.0 [8.5-11.7]
6-9	79	3.2 [2.4-4.4]
10+	57	2.6 [1.8-3.8]

Columns may not sum to 100% due to non-response.
*Respondents were asked, “Are you Spanish, Hispanic, or Latino?” and then, “Please indicate what you consider your race to be. We appreciate your effort to describe your background using these US Census Bureau categories.” Response options were White, Black or African American, American Indian or Alaska Native, Asian, Native Hawaiian or other Pacific Islander, and Some other race. Respondents could select all answers that apply. American Indian or Alaska Native, Native Hawaiian or other Pacific Islander, Some other race, and those who selected more than one race were combined by Ipsos into an Other & 2+ races category.

© 2021 Kravitz-Wirtz N et al. *JAMA Network Open.*

eAppendix 4. Percentage of Respondents Worried About Intimate Partner Violence Happening to Them Before and During the COVID-19 Pandemic, Among Those Worried About a Violent Event Happening to Them, 2020 California Safety and Well-being Survey

	Before the pandemic						During the pandemic					
	No		Yes		Total		No		Yes		Total	
	n	% [95% CI]	n	% [95% CI]	n	%	n	% [95% CI]	n	% [95% CI]	n	%
Total	1881	90 [87.9-91.9]	179	9.7 [7.9-11.8]	2,069	100	1884	90.7 [88.6-92.4]	176	9 [7.3-11.1]	2,069	100
Education												
Less than high school	102	78.5 [69.2-85.6]	29	21.5 [14.4-30.8]	131	100	100	79.4 [70.1-86.4]	25	20 [13.1-29.3]	126	100
High school	255	94.5 [91.3-96.6]	24	5.5 [3.4-8.7]	279	100	257	94.5 [91.4-96.5]	26	5.5 [3.5-8.6]	283	100
Some college	628	92.2 [89.3-94.4]	50	7 [4.9-9.9]	684	100	643	93 [90.3-95.1]	48	6.5 [4.5-9.2]	695	100
Bachelor's degree	481	89.6 [84.0-93.3]	49	10.3 [6.6-15.9]	531	100	486	91.2 [86.4-94.5]	48	8.6 [5.4-13.4]	536	100
Advanced degree	415	92.7 [87.1-96.0]	27	7.1 [3.8-12.7]	444	100	398	91.8 [85.8-95.4]	29	8 [4.4-14.0]	429	100
Gender												
Male	898	84.6 [80.7-87.8]	134	15.3 [12.0-19.1]	1,036	100	905	84.8 [81.0-88.0]	135	15 [11.9-18.8]	1,045	100
Female	983	94.7 [92.2-96.4]	45	4.9 [3.3-7.4]	1,033	100	979	95.8 [93.7-97.2]	41	3.8 [2.4-5.9]	1,024	100
Race/ethnicity												
White, Non-Latinx	1004	93.5 [90.7-95.5]	58	6 [4.0-8.7]	1,068	100	1012	92.9 [89.8-95.1]	61	6.7 [4.5-9.8]	1,078	100
Black, Non-Latinx	90	96.6 [92.0-98.6]	5	3.1 [1.2-7.9]	96	100	80	94.3 [88.1-97.4]	7	5.4 [2.4-11.6]	88	100
Asian, Non-Latinx	148	87.7 [79.8-92.8]	16	12.3 [7.2-20.2]	164	100	151	89.4 [82.3-93.9]	16	10.6 [6.1-17.7]	167	100
Other & 2+ races, Non-Latinx	42	97.2 [90.4-99.2]	3	2.8 [0.8-9.6]	45	100	44	98.7 [94.4-99.7]	2	1.3 [0.3-5.6]	46	100
Latinx	597	86.1 [81.8-89.4]	97	13.8 [10.4-18.0]	696	100	597	87.9 [83.9-90.9]	90	11.8 [8.8-15.8]	690	100
Income												
Less than $25,000	303	88 [81.2-92.6]	39	11.7 [7.2-18.5]	343	100	297	88.2 [81.4-92.8]	36	11.1 [6.7-18.0]	335	100
$25,000-$59,999	512	89.2 [84.8-92.4]	54	10.8 [7.5-15.2]	567	100	527	89.9 [85.9-92.9]	54	10 [7.0-14.1]	582	100
$60,000-$99,999	484	91.7 [87.9-94.4]	39	7.6 [5.0-11.5]	528	100	481	91.7 [87.7-94.4]	39	7.9 [5.2-11.9]	524	100
$100,000 or more	582	90.5 [86.2-93.6]	47	9.2 [6.2-13.5]	631	100	579	91.7 [87.7-94.5]	47	8.1 [5.3-12.1]	628	100
Age												
18-29	163	95.4 [89.9-98.0]	9	4.6 [2.0-10.1]	172	100	156	95.7 [90.1-98.2]	9	4.3 [1.8-9.9]	165	100
30-44	359	88 [83.1-91.7]	46	11.7 [8.1-16.6]	407	100	351	90.3 [85.7-93.5]	40	9.6 [6.4-14.1]	392	100
45-59	483	87.4 [82.6-91.0]	61	12.6 [9.0-17.4]	544	100	505	88.6 [84.2-91.9]	59	11.4 [8.1-15.8]	564	100
60+	876	91.2 [87.5-93.9]	63	7.9 [5.3-11.6]	946	100	872	89.8 [85.8-92.7]	68	9.1 [6.3-13.0]	948	100
Household gun ownership												

© 2021 Kravitz-Wirtz N et al. *JAMA Network Open.*

No guns in home	1349	90.1 [87.5-92.2]	123	9.7 [7.6-12.3]	1,478	100	1324	90.4 [87.9-92.4]	124	9.4 [7.4-11.9]	1,454	100
Gun owner	301	89.4 [83.0-93.5]	29	9.7 [5.7-16.0]	333	100	326	91.4 [85.5-95.0]	26	7.7 [4.3-13.5]	355	100
Non-owner living with owner	149	88.7 [77.8-94.6]	18	11.3 [5.4-22.2]	167	100	147	89.7 [78.0-95.5]	16	10.3 [4.5-22.0]	163	100
Acquired firearms due to the coronavirus pandemic												
Non-gun owner	1580	90.1 [87.8-92.1]	150	9.7 [7.7-12.1]	1,736	100	1558	90.6 [88.3-92.4]	150	9.3 [7.4-11.5]	1,714	100
Gun owner, no purchase	297	89.6 [83.1-93.8]	27	9.4 [5.4-15.9]	327	100	320	91.6 [85.6-95.2]	24	7.5 [4.1-13.5]	347	100
Gun owner, purchase	4	76 [33.7-95.2]	2	24 [4.8-66.3]	6	100	6	80.5 [42.4-95.8]	2	19.5 [4.2-57.6]	8	100

See footnotes at the end of eAppendix 18.

© 2021 Kravitz-Wirtz N et al. *JAMA Network Open.*

eAppendix 5. Percentage of Respondents Willing to Ask for Help From a Hotline, Among Those Worried About Intimate Partner Violence Happening to Them, 2020 California Safety and Well-being Survey

	Not at all willing		Somewhat willing		Very willing		Total		Somewhat vs. not all willing	Very vs. not at all willing
	n	% [95% CI]	n	% [95% CI]	n	% [95% CI]	n	%	PD	PD
Total	50	24.6 [16.7-34.5]	68	33.8 [25.3-43.5]	91	41.7 [32.1-51.9]	209	100	9.2	17.1[a]
Education										
Less than high school	5	20.5 [8.1-43.1]	10	30.6 [15.8-51.0]	16	48.8 [29.4-68.7]	31	100	10.1	28.3
High school	7	27.4 [12.3-50.4]	8	33.6 [16.8-56.0]	14	39 [21.5-60.0]	29	100	6.3	11.7
Some college	13	26.4 [13.4-45.3]	18	35 [21.0-52.1]	28	38.7 [24.8-54.7]	59	100	8.6	12.3
Bachelor's degree	17	20.8 [9.4-39.9]	20	41.5 [22.5-63.5]	19	37.7 [18.4-61.8]	56	100	20.7	16.9
Advanced degree	8	37.9 [14.6-68.5]	12	29 [12.7-53.4]	14	33.1 [13.4-61.3]	34	100	-8.9	-4.8
Gender										
Male	43	30 [20.2-42.1]	52	33.9 [24.2-45.1]	65	36.1 [25.9-47.8]	160	100	3.9	6.1
Female	7	8.3 [3.3-19.5]	16	33.4 [17.9-53.6]	26	58.3 [38.5-75.7]	49	100	25.0[a]	49.9[a]
Race/ethnicity										
White, Non-Latinx	29	37.5 [22.4-55.5]	18	20.3 [10.9-34.7]	26	42.2 [25.7-60.7]	73	100	-17.2	4.7
Black, Non-Latinx	2	26.7 [6.1-67.0]	2	27.8 [6.3-68.7]	3	45.4 [15.0-79.8]	7	100	1.1	18.7
Asian, Non-Latinx	7	43.4 [20.8-69.0]	8	33.5 [14.7-59.5]	3	23.1 [7.2-54.0]	18	100	-9.8	-20.3
Other & 2+ races, Non-Latinx	0	0 [NA]	1	20.8 [2.2-75.0]	2	79.2 [25.0-97.8]	3	100	20.8	79.2[a]
Latinx	12	11.3 [4.3-26.6]	39	41.4 [28.5-55.5]	57	47.3 [33.6-61.5]	108	100	30.1[a]	36.0[a]
Income										
Less than $25,000	7	17.6 [5.1-45.5]	12	27.5 [12.7-49.8]	25	54.9 [31.5-76.3]	44	100	10.0	37.4[b]
$25,000-$59,999	14	13.5 [6.5-26.1]	22	39.6 [24.2-57.3]	27	46.9 [30.7-63.7]	63	100	26.1[a]	33.3[a]
$60,000-$99,999	11	24.9 [11.9-44.9]	14	36.8 [19.8-58.0]	20	38.3 [21.7-58.2]	45	100	12.0	13.4
$100,000 or more	18	38.2 [21.7-58.1]	20	30.8 [17.3-48.6]	19	31 [16.0-51.4]	57	100	-7.4	-7.3
Age										
18-29	1	3.9 [0.5-25.3]	5	31.6 [9.4-67.1]	4	64.6 [29.5-88.8]	10	100	27.7[b]	60.7[a]
30-44	8	21.6 [9.7-41.2]	22	36.4 [21.4-54.7]	19	42 [25.1-61.0]	49	100	14.8	20.5
45-59	19	31.7 [17.5-50.4]	21	36.6 [22.5-53.3]	30	31.7 [19.6-46.9]	70	100	4.8	0.0
60+	22	26 [14.3-42.6]	20	26.4 [14.3-43.5]	38	47.6 [30.6-65.1]	80	100	0.3	21.5

© 2021 Kravitz-Wirtz N et al. *JAMA Network Open.*

Household gun ownership										
No guns in home	33	25 [15.9-37.1]	49	35.4 [25.3-47.1]	65	39.6 [28.8-51.4]	147	100	10.4	14.6
Gun owner	10	19.3 [8.4-38.3]	10	36.9 [16.8-63.0]	11	43.8 [19.8-71.1]	31	100	17.6	24.5
Non-owner living with owner	3	22.7 [4.1-67.0]	7	22.8 [7.6-51.7]	10	54.5 [21.7-83.7]	20	100	0.1	31.8
Acquired firearms due to the coronavirus pandemic										
Non-gun owner	40	25.3 [16.7-36.4]	58	33.3 [24.3-43.8]	80	41.4 [31.2-52.4]	178	100	8.1	16.1
Gun owner, no purchase	8	15.7 [6.2-34.3]	10	38.6 [17.3-65.3]	11	45.8 [20.8-73.1]	29	100	22.9	30.1
Gun owner, purchase	2	100 [NA]	0	0 [NA]	0	0 [NA]	2	100	-100.0	-100.0

See footnotes at the end of eAppendix 18.

© 2021 Kravitz-Wirtz N et al. *JAMA Network Open.*

eAppendix 6. Percentage of Respondents Willing to Ask for Help From Family, Among Those Worried About Intimate Partner Violence Happening to Them, 2020 California Safety and Well-being Survey

	Not at all willing		Somewhat willing		Very willing		Total		Somewhat vs. not all willing	Very vs. not at all willing
	n	% [95% CI]	n	% [95% CI]	n	% [95% CI]	n	% [95% CI]	PD	PD
Total	20	13.2 [7.2-22.9]	75	35.3 [26.5-45.1]	114	51.6 [41.5-61.5]	209	100	22.1[a]	38.4[a]
Education										
Less than high school	6	23.7 [10.1-46.0]	10	33.5 [17.8-53.9]	15	42.8 [24.3-63.6]	31	100	9.8	19.2
High school	3	10.2 [2.4-35.0]	11	41.1 [22.8-62.3]	15	48.7 [28.8-69.0]	29	100	30.9[a]	38.5[a]
Some college	3	5.1 [1.4-16.5]	21	39.5 [24.5-56.9]	35	55.4 [38.6-71.0]	59	100	34.4[a]	50.3[a]
Bachelor's degree	6	11.7 [3.4-33.4]	22	27.3 [14.7-44.9]	28	61 [40.5-78.3]	56	100	15.6	49.3[a]
Advanced degree	2	0.9 [0.2-3.8]	11	37.3 [14.4-67.8]	21	61.8 [31.8-84.9]	34	100	36.4[a]	61.0[a]
Gender									0.0	0.0
Male	15	14.4 [7.1-26.9]	60	36.7 [26.6-48.1]	85	48.9 [37.6-60.4]	160	100	22.3[a]	34.6[a]
Female	5	9.7 [3.4-24.8]	15	31 [16.0-51.3]	29	59.4 [39.3-76.7]	49	100	21.3[b]	49.7[a]
Race/ethnicity										
White, Non-Latinx	5	11.9 [3.1-36.0]	27	27.9 [16.1-43.9]	41	60.2 [42.2-75.8]	73	100	16.0	48.3[a]
Black, Non-Latinx	0	0 [NA]	3	33.7 [9.7-70.5]	4	66.3 [29.5-90.3]	7	100	33.7[b]	66.3[a]
Asian, Non-Latinx	2	9.9 [2.3-34.2]	8	38.7 [17.7-65.0]	8	51.4 [26.9-75.2]	18	100	28.8[b]	41.4[a]
Other & 2+ races, Non-Latinx	0	0 [NA]	1	20.8 [2.2-75.0]	2	79.2 [25.0-97.8]	3	100	20.8	79.2[a]
Latinx	13	15.9 [7.3-31.1]	36	38.1 [25.7-52.4]	59	46 [32.4-60.2]	108	100	22.3[a]	30.1[a]
Income										
Less than $25,000	7	30.3 [11.6-58.9]	13	32 [15.2-55.3]	24	37.7 [18.3-62.1]	44	100	1.7	7.4
$25,000-$59,999	4	9.8 [2.5-31.0]	24	41.7 [26.1-59.2]	35	48.5 [32.3-65.1]	63	100	31.9[a]	38.8[a]
$60,000-$99,999	4	9 [2.8-25.8]	18	35.3 [19.0-55.9]	23	55.7 [36.0-73.7]	45	100	26.3[a]	46.6[a]
$100,000 or more	5	8 [2.5-23.0]	20	31.6 [17.5-50.2]	32	60.4 [41.8-76.4]	57	100	23.6[a]	52.4[a]
Age										
18-29	1	29.6 [4.8-77.7]	4	29.5 [8.4-65.5]	5	40.9 [12.6-76.9]	10	100	-0.1	11.3
30-44	7	17.8 [7.2-37.7]	16	30.7 [16.7-49.5]	26	51.5 [33.5-69.2]	49	100	12.9	33.7[a]
45-59	7	9 [3.2-22.8]	27	42 [27.0-58.7]	36	49 [33.1-65.1]	70	100	33.1[a]	40.0[a]
60+	5	6.4 [1.8-20.1]	28	34.4 [20.5-51.6]	47	59.2 [41.9-74.5]	80	100	28.0[a]	52.9[a]

© 2021 Kravitz-Wirtz N et al. *JAMA Network Open.*

Household gun ownership										
No guns in home	15	15.8 [8.3-28.3]	50	34 [24.1-45.6]	82	50.2 [38.6-61.7]	147	100	18.1[a]	34.3[a]
Gun owner	1	5.5 [0.8-31.1]	14	40.6 [19.4-66.0]	16	53.9 [28.8-77.2]	31	100	35.0[a]	48.4[a]
Non-owner living with owner	1	0.7 [0.1-5.3]	8	39.8 [13.1-74.4]	11	59.5 [25.2-86.5]	20	100	39.1[a]	58.9[a]
Acquired firearms due to the coronavirus pandemic										
Non-gun owner	19	14.2 [7.6-25.1]	61	34.5 [25.2-45.2]	98	51.3 [40.5-61.9]	178	100	20.3[a]	37.0[a]
Gun owner, no purchase	1	5.8 [0.8-32.2]	13	39.6 [18.2-66.0]	15	54.6 [28.6-78.3]	29	100	33.8[a]	48.8[a]
Gun owner, purchase	0	0 [NA]	1	61.7 [9.1-96.3]	1	38.3 [3.7-90.9]	2	100	61.8[b]	38.2

See footnotes at the end of eAppendix 18.

© 2021 Kravitz-Wirtz N et al. *JAMA Network Open.*

eAppendix 7. Percentage of Respondents Willing to Ask for Help From Law Enforcement, Among Those Worried About Intimate Partner Violence Happening to Them, 2020 California Safety and Well-being Survey

	Not at all willing		Somewhat willing		Very willing		Total		Somewhat vs. not all willing	Very vs. not at all willing
	n	% [95% CI]	n	% [95% CI]	n	% [95% CI]	n	% [95% CI]	PD	PD
Total	22	10.5 [5.4-19.5]	85	42.9 [33.4-52.9]	102	46.6 [36.8-56.7]	209	100	32.4[a]	36.1[a]
Education										
Less than high school	4	16.3 [5.5-39.7]	14	44.2 [25.8-64.3]	13	39.4 [21.6-60.7]	31	100	27.9[b]	23.1
High school	1	6.1 [0.9-32.6]	9	38 [20.1-59.9]	19	55.9 [34.7-75.1]	29	100	31.9[a]	49.8[a]
Some college	3	6.7 [2.1-19.6]	22	31.8 [19.0-48.1]	34	61.4 [45.0-75.6]	59	100	25.1[a]	54.7[a]
Bachelor's degree	10	8.4 [3.6-18.3]	25	52.1 [30.6-72.8]	21	39.5 [20.0-63.0]	56	100	43.6[a]	31.1[a]
Advanced degree	4	7.7 [1.7-28.8]	15	56 [29.5-79.4]	15	36.3 [15.5-63.9]	34	100	48.2[a]	28.6[b]
Gender										
Male	15	10.9 [4.8-23.0]	66	43.7 [32.8-55.3]	79	45.4 [34.4-56.9]	160	100	32.8[a]	34.5[a]
Female	7	9.3 [3.9-20.8]	19	40.5 [23.2-60.5]	23	50.2 [30.7-69.7]	49	100	31.1[a]	40.9[a]
Race/ethnicity										
White, Non-Latinx	10	18.4 [7.3-39.1]	26	26.7 [15.4-42.1]	37	55 [37.4-71.4]	73	100	8.3	36.6[a]
Black, Non-Latinx	0	0 [NA]	3	53.7 [20.0-84.3]	4	46.3 [15.7-80.0]	7	100	53.7[a]	46.3[a]
Asian, Non-Latinx	1	3.5 [0.5-21.9]	10	51.7 [27.1-75.6]	7	44.7 [21.7-70.3]	18	100	48.2[a]	41.2[a]
Other & 2+ races, Non-Latinx	0	0 [NA]	2	72.6 [18.3-96.9]	1	27.4 [3.1-81.7]	3	100	72.6[a]	27.4
Latinx	11	9.6 [3.4-24.4]	44	47.2 [33.5-61.3]	53	43.2 [30.0-57.5]	108	100	37.6[a]	33.6[a]
Income										
Less than $25,000	4	14 [3.1-45.4]	15	36.2 [18.1-59.3]	25	49.8 [27.0-72.7]	44	100	22.2	35.8[b]
$25,000-$59,999	3	2.2 [0.6-8.2]	28	52.3 [35.7-68.4]	32	45.5 [29.8-62.1]	63	100	50.1[a]	43.2[a]
$60,000-$99,999	8	16.2 [6.8-33.9]	17	36.2 [19.7-56.7]	20	47.6 [28.9-67.0]	45	100	20.0	31.3[a]
$100,000 or more	7	12.5 [3.7-34.7]	25	42.3 [25.7-60.9]	25	45.2 [27.6-64.1]	57	100	29.8[a]	32.7[a]
Age										
18-29	2	6 [1.2-24.4]	3	25.6 [6.6-62.6]	5	68.4 [32.9-90.6]	10	100	19.7	62.5[a]
30-44	4	10.3 [3.0-29.8]	25	50.8 [32.8-68.6]	20	38.9 [22.7-57.9]	49	100	40.6[a]	28.6[a]
45-59	7	11.3 [3.1-33.6]	27	44.3 [29.0-60.8]	36	44.4 [29.3-60.5]	70	100	33.0[a]	33.0[a]
60+	9	11.2 [4.3-26.0]	30	34.7 [20.9-51.8]	41	54.1 [36.9-70.4]	80	100	23.5[a]	42.9[a]

© 2021 Kravitz-Wirtz N et al. *JAMA Network Open.*

Household gun ownership										
No guns in home	16	11.3 [5.1-23.1]	65	46.8 [35.5-58.5]	66	41.9 [30.9-53.7]	147	100	35.5[a]	30.6[a]
Gun owner	3	8.5 [2.0-30.0]	8	23.6 [8.8-49.8]	20	67.9 [42.1-86.0]	31	100	15.2	59.5[a]
Non-owner living with owner	2	8.5 [1.5-35.5]	11	48.2 [17.7-80.1]	7	43.4 [13.9-78.4]	20	100	39.7[b]	34.9
Acquired firearms due to the coronavirus pandemic										
Non-gun owner	19	10.8 [5.2-20.9]	77	45.5 [35.1-56.3]	82	43.7 [33.4-54.7]	178	100	34.7	33.0
Gun owner, no purchase	3	8.8 [2.0-31.2]	8	24.7 [9.1-51.7]	18	66.5 [39.9-85.6]	29	100	15.9	57.6
Gun owner, purchase	0	0 [NA]	0	0 [NA]	2	100 [NA]	2	100	0.0	100.0

See footnotes at the end of eAppendix 18.

© 2021 Kravitz-Wirtz N et al. *JAMA Network Open.*

eAppendix 8. Percentage of Respondents Worried About Homicide Happening to Them Before and During the COVID-19 Pandemic, 2020 California Safety and Well-being Survey

	Before the pandemic						During the pandemic						Total		During - Before		
	Not worried		Somewhat worried		Very worried		Not worried		Somewhat worried		Very worried				Not	Somewhat	Very
	n	% [95% CI]	n	% [95% CI]	n	% [95% CI]	n	% [95% CI]	n	% [95% CI]	n	% [95% CI]	n	%	PD	PD	PD
Total	1821	54.7 [51.8-57.5]	784	32.1 [29.5-34.8]	234	11.9 [10.1-14.1]	1728	51.2 [48.4-54.0]	792	32.5 [29.9-35.2]	320	15.1 [13.1-17.4]	2,870	100	-3.5[b]	0.4	3.2[a]
Education																	
Less than high school	46	28 [20.9-36.5]	66	44.6 [35.9-53.6]	43	24.9 [18.0-33.4]	47	29 [21.7-37.6]	59	39.4 [31.1-48.5]	46	28.5 [21.1-37.2]	158	100	0.9	-5.1	3.6
High school	167	46.6 [39.9-53.4]	113	31.5 [25.4-38.2]	73	20 [15.0-26.2]	153	40.7 [34.2-47.5]	117	34.8 [28.5-41.7]	87	23 [17.7-29.3]	364	100	-5.9	3.4	2.9
Some college	601	61.5 [57.0-65.8]	265	29.8 [25.9-34.1]	65	7.8 [5.6-10.8]	576	59 [54.5-63.3]	263	29.8 [25.9-34.1]	93	10.7 [8.2-13.9]	938	100	-2.5	0.0	2.9
Bachelor's degree	547	63.4 [57.7-68.8]	190	30.2 [25.2-35.7]	34	5.5 [3.0-9.8]	509	58.6 [52.9-64.1]	203	30.9 [25.8-36.4]	58	9.7 [6.6-14.2]	775	100	-4.8	0.7	4.3[b]
Advanced degree	460	69 [62.5-74.8]	150	27 [21.5-33.4]	19	3.6 [1.8-7.2]	443	63.5 [56.7-69.7]	150	29.6 [23.6-36.5]	36	6 [3.7-9.7]	635	100	-5.5	2.6	2.4
Gender																	
Male	988	56 [51.9-60.1]	381	30.1 [26.4-34.1]	121	12.7 [9.9-16.2]	944	52.7 [48.6-56.8]	399	32.2 [28.4-36.3]	148	13.8 [11.1-17.2]	1,506	100	-3.3	2.1	1.1
Female	833	53.4 [49.6-57.2]	403	33.9 [30.3-37.7]	113	11.2 [8.8-14.1]	784	49.7 [45.9-53.5]	393	32.7 [29.2-36.4]	172	16.3 [13.5-19.6]	1,364	100	-3.7	-1.2	5.1[a]
Race/ethnicity																	
White, Non-Latinx	1226	73.7 [70.2-76.9]	322	20.2 [17.4-23.2]	55	5.3 [3.6-7.8]	1169	68.2 [64.5-71.7]	364	25.2 [22.1-28.7]	72	5.8 [4.1-8.1]	1,615	100	-5.5[a]	5.1[a]	0.5
Black, Non-Latinx	65	43.2 [32.0-55.1]	48	44.9 [33.2-57.1]	13	11.8 [6.0-22.0]	66	44.8 [33.4-56.8]	48	43.4 [31.8-55.7]	12	11.2 [5.6-21.4]	127	100	1.7	-1.5	-0.6
Asian, Non-Latinx	103	48.9 [40.5-57.4]	86	41.8 [33.7-50.4]	19	9.3 [5.3-15.7]	102	44.7 [36.5-53.2]	73	39.9 [31.8-48.6]	33	15.4 [10.2-22.6]	208	100	-4.2	-2.0	6.1
Other & 2+ races, Non-Latinx	47	62.4 [44.7-77.3]	20	31.7 [17.8-50.0]	3	3.6 [1.0-12.5]	50	68.3 [50.3-82.1]	16	22.4 [11.4-39.1]	5	9.3 [2.5-29.5]	71	100	5.9	-9.4	5.7
Latinx	380	35.3 [30.9-40.0]	308	40.4 [35.5-45.4]	144	21.9 [17.8-26.6]	341	32.7 [28.4-37.4]	291	37.3 [32.6-42.2]	198	27.5 [23.1-32.3]	849	100	-2.6	-3.1	5.6[b]
Income																	
Less than $25,000	188	34.4 [28.2-41.2]	149	40.4 [33.5-47.7]	84	21.6 [16.2-28.1]	199	39.7 [33.0-46.8]	124	34.3 [27.8-41.5]	96	22.4 [16.9-29.1]	431	100	5.4	-6.1	0.9
$25,000-$59,999	435	43.6 [38.4-48.9]	222	38.2 [32.9-43.8]	79	17.6 [13.4-22.7]	400	38.4 [33.5-43.4]	237	39.3 [34.0-44.9]	101	21.6 [17.1-26.9]	744	100	-5.2	1.0	4.0
$60,000-$99,999	493	55.1 [49.4-60.7]	212	33.6 [28.4-39.2]	46	10.2 [6.8-14.9]	475	51.9 [46.3-57.5]	198	31 [25.9-36.6]	78	16.4 [12.3-21.5]	757	100	-3.2	-2.6	6.2[a]
$100,000 or more	705	69.2 [64.5-73.6]	201	24.1 [20.2-28.5]	25	5.7 [3.5-9.2]	654	63.2 [58.3-67.7]	233	28.4 [24.2-33.0]	45	7.5 [5.0-11.0]	938	100	-6.1[b]	4.3	1.8

© 2021 Kravitz-Wirtz N et al. *JAMA Network Open.*

Age																	
18-29	105	51.3 [42.9-59.6]	71	35.1 [27.5-43.5]	21	12.4 [7.6-19.4]	102	47.9 [39.6-56.3]	63	35.3 [27.6-43.8]	33	16 [10.7-23.3]	200	100	-3.4	0.2	3.6
30-44	248	47.3 [41.5-53.1]	187	35.2 [29.9-40.9]	69	16.3 [12.2-21.5]	231	42.7 [37.0-48.5]	181	37.4 [31.9-43.2]	91	18.6 [14.4-23.7]	511	100	-4.6	2.2	2.3
45-59	428	51 [45.9-56.1]	225	34.8 [30.0-40.0]	76	12.1 [9.1-16.1]	394	48.1 [43.1-53.2]	231	33.2 [28.5-38.2]	104	17 [13.3-21.5]	738	100	-2.9	-1.6	4.9[b]
60+	1040	68.3 [64.4-71.9]	301	24.2 [20.9-27.9]	68	6.8 [4.8-9.4]	1001	65.4 [61.5-69.0]	317	24.8 [21.6-28.3]	92	9 [6.7-12.0]	1,421	100	-2.9	0.6	2.2
Household gun ownership																	
No guns in home	1208	51.2 [47.9-54.6]	570	34.2 [31.0-37.5]	192	13.4 [11.1-16.0]	1154	48 [44.7-51.3]	563	34 [30.8-37.3]	255	16.8 [14.3-19.7]	1,989	100	-3.2	-0.2	3.5[b]
Gun owner	387	68.4 [61.6-74.4]	124	25.8 [20.3-32.1]	16	5.3 [2.6-10.6]	361	64 [57.1-70.3]	138	28 [22.2-34.7]	28	7.9 [4.7-13.2]	529	100	-4.4	2.2	2.7
Non-owner living with owner	149	61.2 [51.1-70.5]	57	26.5 [18.9-35.9]	12	12 [6.1-22.4]	142	59.1 [49.1-68.4]	52	24.4 [17.1-33.5]	24	16.2 [9.5-26.2]	219	100	-2.1	-2.1	4.1
Acquired firearms due to the coronavirus pandemic																	
Non-gun owner	1434	52.2 [49.1-55.3]	660	33.2 [30.3-36.2]	218	13.1 [11.0-15.6]	1367	48.9 [45.8-51.9]	654	33.3 [30.4-36.3]	292	16.4 [14.1-19.0]	2,341	100	-3.3	0.1	3.3
Gun owner, no purchase	378	67.7 [60.8-73.9]	123	26.3 [20.7-32.8]	16	5.4 [2.6-10.9]	354	63.5 [56.4-70.0]	137	28.6 [22.7-35.4]	26	7.8 [4.5-13.2]	519	100	-4.2	2.3	2.4
Gun owner, purchase	9	95.5 [72.0-99.4]	1	4.5 [0.6-28.0]	0	0 [NA]	7	84.6 [56.0-96.0]	1	3.6 [0.5-23.7]	2	11.7 [2.5-40.9]	10	100	-10.9	-0.9	11.7

See footnotes at the end of eAppendix 18.

© 2021 Kravitz-Wirtz N et al. *JAMA Network Open.*

eAppendix 9. Percentage of Respondents Worried About Suicide Happening to Them Before and During the COVID-19 Pandemic, 2020 California Safety and Well-being Survey

	Before the pandemic						During the pandemic						Total		During - Before		
	Not worried		Somewhat worried		Very worried		Not worried		Somewhat worried		Very worried				Not	Somewhat	Very
	n	% [95% CI]	n	% [95% CI]	n	% [95% CI]	n	% [95% CI]	n	% [95% CI]	n	% [95% CI]	n	%	PD	PD	PD
Total	2351	74.6 [71.9-77.1]	335	16.6 [14.5-19.1]	162	7.9 [6.4-9.7]	2293	71 [68.3-73.7]	353	18.7 [16.4-21.2]	199	9.1 [7.5-10.9]	2,870	100	-3.5[b]	2.1	1.2
Education																	
Less than high school	79	52.2 [43.3-61.0]	45	31 [23.3-39.9]	31	15.1 [9.9-22.3]	70	43.9 [35.4-52.9]	51	36.3 [28.1-45.3]	31	16.7 [11.1-24.4]	158	100	-8.3	5.3	1.6
High school	257	70.2 [63.6-76.1]	55	17 [12.4-22.9]	45	11.4 [7.8-16.5]	241	65.9 [59.1-72.0]	62	19.9 [14.9-26.1]	53	12.5 [8.6-17.6]	364	100	-4.4	2.9	1.0
Some college	781	78.4 [73.9-82.3]	110	14.5 [11.2-18.5]	42	6.5 [4.4-9.6]	775	78 [73.7-81.8]	104	14.6 [11.3-18.6]	54	6.8 [4.8-9.5]	938	100	-0.4	0.1	0.2
Bachelor's degree	664	80.6 [75.3-85.0]	80	14 [10.2-19.0]	30	4.9 [2.9-8.1]	650	77.1 [71.6-81.8]	82	14.1 [10.5-18.6]	41	8.3 [5.2-13.0]	775	100	-3.5	0.1	3.4
Advanced degree	570	89 [84.1-92.5]	45	8.5 [5.3-13.4]	14	1.8 [0.9-3.5]	557	84.6 [78.8-89.0]	54	12.8 [8.6-18.7]	20	1.9 [1.1-3.5]	635	100	-4.4	4.3	0.2
Gender																	
Male	1261	75.1 [71.0-78.8]	163	17.2 [13.9-21.0]	69	6.6 [4.8-9.1]	1224	71.9 [67.8-75.7]	168	18 [14.8-21.8]	101	8.6 [6.5-11.3]	1,506	100	-3.2	0.8	2.0
Female	1090	74.1 [70.4-77.4]	172	16.1 [13.4-19.3]	93	9 [6.9-11.7]	1069	70.3 [66.4-73.8]	185	19.3 [16.2-22.8]	98	9.5 [7.3-12.2]	1,364	100	-3.8	3.2	0.4
Race/ethnicity																	
White, Non-Latinx	1444	84.6 [81.2-87.5]	125	10.8 [8.4-13.8]	37	3.8 [2.4-6.0]	1434	84.8 [81.6-87.5]	128	10.5 [8.3-13.2]	46	4 [2.6-6.2]	1,615	100	0.2	-0.3	0.3
Black, Non-Latinx	102	69.6 [56.3-80.2]	19	22.3 [13.2-35.2]	6	8.2 [3.0-20.4]	104	70.1 [56.7-80.7]	19	24.8 [15.0-38.1]	4	5.1 [1.4-16.3]	127	100	0.5	2.5	-3.1
Asian, Non-Latinx	162	77.3 [69.4-83.7]	33	17 [11.4-24.7]	12	5.3 [2.7-10.2]	152	72.2 [63.8-79.2]	38	19.7 [13.6-27.6]	18	8.2 [4.6-14.1]	208	100	-5.2	2.6	2.8
Other & 2+ races, Non-Latinx	60	84 [67.5-93.0]	10	14.9 [6.2-31.7]	1	1.1 [0.1-7.3]	57	79.7 [63.4-89.9]	13	19.2 [9.3-35.6]	1	1.1 [0.1-7.3]	71	100	-4.3	4.3	0.0
Latinx	583	61.2 [56.1-66.1]	148	22.7 [18.6-27.5]	106	14.5 [11.3-18.5]	546	53.3 [48.2-58.3]	155	27.1 [22.6-32.1]	130	16.9 [13.3-21.1]	849	100	-7.9[a]	4.4	2.3
Income																	
Less than $25,000	274	54 [46.7-61.1]	85	27.2 [21.0-34.5]	62	15.3 [10.8-21.3]	267	51.8 [44.6-58.9]	80	26.9 [20.8-33.9]	74	17.6 [12.6-23.9]	431	100	-2.1	-0.3	2.3
$25,000-$59,999	597	70.6 [65.0-75.7]	95	18.8 [14.6-24.0]	49	10.3 [7.2-14.6]	575	65.3 [59.5-70.6]	101	21.6 [17.0-27.1]	61	11.9 [8.6-16.3]	744	100	-5.4	2.8	1.6
$60,000-$99,999	636	76.3 [70.7-81.1]	88	16.2 [12.1-21.3]	31	7.4 [4.7-11.4]	628	73.7 [68.0-78.8]	86	16.9 [12.7-22.2]	41	9.1 [6.1-13.5]	757	100	-2.5	0.7	1.8
$100,000 or more	844	84 [79.7-87.6]	67	11.4 [8.3-15.4]	20	3.8 [2.2-6.5]	823	80.5 [76.0-84.4]	86	14.7 [11.3-18.9]	23	3.9 [2.2-6.7]	938	100	-3.5	3.4	0.1

© 2021 Kravitz-Wirtz N et al. *JAMA Network Open.*

Age																	
18-29	137	69 [60.8-76.3]	39	20.3 [14.2-28.2]	21	9.8 [6.0-15.5]	131	67.5 [59.3-74.8]	40	17.4 [12.1-24.4]	26	13.8 [8.9-20.8]	200	100	-1.5	-2.8	4.0
30-44	356	65.3 [59.4-70.7]	100	24 [19.2-29.6]	48	9.5 [6.5-13.7]	328	59.8 [53.9-65.4]	117	28.1 [23.0-33.7]	57	10.7 [7.5-15.0]	511	100	-5.5	4.1	1.2
45-59	578	73.6 [68.5-78.0]	97	15.9 [12.3-20.4]	57	9.3 [6.7-12.8]	561	67.9 [62.6-72.8]	103	20.9 [16.5-26.1]	65	9.3 [6.8-12.5]	738	100	-5.7	5.0	0.0
60+	1280	88.9 [86.0-91.2]	99	7.1 [5.5-9.3]	36	3.6 [2.1-6.1]	1273	88.4 [85.5-90.8]	93	7.1 [5.4-9.3]	51	4.2 [2.7-6.6]	1,421	100	-0.5	0.0	0.6
Household gun ownership																	
No guns in home	1588	71.8 [68.5-74.9]	252	18 [15.4-21.0]	136	9.4 [7.5-11.7]	1531	67.2 [63.8-70.4]	275	21.2 [18.4-24.3]	166	10.4 [8.4-12.8]	1,989	100	-4.6[a]	3.2	1.0
Gun owner	487	90.5 [85.2-94.0]	34	8.1 [4.8-13.4]	6	1 [0.4-2.6]	483	88.3 [82.5-92.4]	33	8.6 [5.1-14.0]	12	2.9 [1.2-6.8]	529	100	-2.2	0.5	1.9
Non-owner living with owner	180	78.6 [68.7-86.0]	27	12.2 [6.9-20.6]	12	9.2 [4.5-18.2]	183	78.9 [69.0-86.2]	23	11.5 [6.4-19.7]	13	9.6 [4.7-18.7]	219	100	0.3	-0.7	0.4
Acquired firearms due to the coronavirus pandemic																	
Non-gun owner	1864	71.7 [68.7-74.5]	301	18.2 [15.7-20.9]	156	9.1 [7.4-11.2]	1810	67.9 [64.9-70.9]	320	20.5 [17.9-23.3]	187	10.2 [8.3-12.3]	2,341	100	-3.8	2.3	1.0
Gun owner, no purchase	478	90.3 [84.9-93.9]	33	8.2 [4.8-13.6]	6	1 [0.4-2.7]	474	88.1 [82.2-92.3]	33	8.8 [5.2-14.3]	11	2.9 [1.2-6.9]	519	100	-2.2	0.6	1.8
Gun owner, purchase	9	95.5 [72.0-99.4]	1	4.5 [0.6-28.0]	0	0 [NA]	9	95.5 [72.0-99.4]	0	0 [NA]	1	4.5 [0.6-28.0]	10	100	0.0	-4.5	4.5

See footnotes at the end of eAppendix 18.

© 2021 Kravitz-Wirtz N et al. *JAMA Network Open.*

eAppendix 10. Percentage of Respondents Worried About a Mass Shooting Happening to Them Before and During the COVID-19 Pandemic, 2020 California Safety and Well-being Survey

	Before the pandemic						During the pandemic						Total		During - Before		
	Not worried		Somewhat worried		Very worried		Not worried		Somewhat worried		Very worried				Not	Somewhat	Very
	n	% [95% CI]	n	% [95% CI]	n	% [95% CI]	n	% [95% CI]	n	% [95% CI]	n	% [95% CI]	n	%	PD	PD	PD
Total	1347	39.1 [36.5-41.7]	1086	41.2 [38.4-44.0]	412	18.8 [16.6-21.2]	1471	43.2 [40.5-46.0]	956	37.4 [34.7-40.1]	410	18 [15.8-20.3]	2,870	100	4.2[a]	-3.8[b]	-0.8
Education																	
Less than high school	40	26.7 [19.5-35.2]	58	37.4 [29.3-46.4]	58	34.3 [26.4-43.2]	37	23.4 [16.7-31.7]	64	41.1 [32.7-50.1]	54	32.2 [24.5-41.0]	158	100	-3.3	3.7	-2.1
High school	131	34.9 [28.8-41.6]	133	40 [33.4-47.0]	93	23.5 [18.2-29.8]	128	36.5 [30.2-43.4]	126	35.5 [29.3-42.4]	103	26.3 [20.8-32.7]	364	100	1.6	-4.5	2.8
Some college	472	46 [41.6-50.4]	340	39.8 [35.4-44.3]	119	13.6 [10.8-16.9]	479	47.5 [43.0-51.9]	328	37.6 [33.4-42.1]	121	13.9 [11.0-17.4]	938	100	1.5	-2.1	0.3
Bachelor's degree	373	37.9 [32.9-43.1]	317	47.3 [41.7-52.9]	80	14 [10.2-18.9]	426	47.3 [41.8-52.8]	262	39.8 [34.3-45.4]	80	12.1 [8.7-16.7]	775	100	9.4[a]	-7.5[b]	-1.8
Advanced degree	331	44.1 [38.0-50.4]	238	42.8 [36.4-49.5]	62	12.8 [8.8-18.2]	401	60 [53.3-66.2]	176	32.3 [26.2-39.1]	52	6.9 [4.8-9.9]	635	100	15.9[a]	-10.5[a]	-5.9[a]
Gender																	
Male	800	44.3 [40.4-48.4]	513	37.2 [33.2-41.3]	181	17.4 [14.3-21.1]	849	47.7 [43.7-51.8]	449	33.2 [29.4-37.3]	192	17.2 [14.2-20.8]	1,506	100	3.4	-4.0	-0.2
Female	547	34.2 [30.8-37.8]	573	44.9 [41.1-48.7]	231	20 [17.0-23.3]	622	39.1 [35.5-42.8]	507	41.2 [37.5-45.0]	218	18.6 [15.7-21.9]	1,364	100	4.9[b]	-3.7	-1.3
Race/ethnicity																	
White, Non-Latinx	929	53.7 [50.0-57.5]	570	37.4 [33.8-41.1]	108	8.2 [6.2-10.7]	1004	58 [54.2-61.7]	485	31.9 [28.5-35.5]	115	9.3 [7.1-12.1]	1,615	100	4.3	-5.5[a]	1.1
Black, Non-Latinx	61	45.9 [34.3-58.0]	42	35.9 [25.4-48.1]	24	18.2 [10.4-29.7]	63	42.5 [31.4-54.5]	46	44 [32.4-56.3]	18	13.5 [7.4-23.3]	127	100	-3.4	8.1	-4.7
Asian, Non-Latinx	70	26.9 [20.4-34.5]	96	53.3 [44.8-61.5]	40	19.3 [13.5-26.8]	91	40.1 [32.2-48.6]	81	43.9 [35.6-52.5]	34	15.3 [10.1-22.3]	208	100	13.2[a]	-9.4	-4.0
Other & 2+ races, Non-Latinx	39	48.6 [32.1-65.5]	22	37.8 [22.4-56.1]	10	13.6 [6.3-26.9]	43	56.3 [39.1-72.1]	19	29.5 [16.8-46.3]	9	14.2 [5.4-32.4]	71	100	7.7	-8.3	0.7
Latinx	248	24.4 [20.5-28.7]	356	41.9 [37.1-46.9]	230	31.9 [27.3-36.8]	270	25.6 [21.6-29.9]	325	40.9 [36.0-45.9]	234	30.7 [26.3-35.5]	849	100	1.2	-1.1	-1.2
Income																	
Less than $25,000	158	32.2 [26.0-39.1]	149	36.7 [30.0-43.9]	116	27.8 [21.9-34.6]	163	32.6 [26.4-39.5]	145	36 [29.4-43.2]	115	28 [22.0-34.9]	431	100	0.4	-0.6	0.2
$25,000-$59,999	339	34.1 [29.3-39.1]	268	40.4 [35.2-45.8]	131	25 [20.2-30.5]	349	34.3 [29.6-39.3]	253	40.8 [35.5-46.4]	129	23.3 [18.9-28.5]	744	100	0.2	0.4	-1.7
$60,000-$99,999	363	39.3 [34.1-44.8]	300	41 [35.6-46.7]	91	19.3 [14.9-24.6]	415	43.8 [38.4-49.3]	237	35.4 [30.2-41.0]	98	19.6 [15.2-24.9]	757	100	4.5	-5.6	0.4
$100,000 or more	487	44.7 [40.1-49.3]	369	43.6 [38.9-48.3]	74	11 [8.2-14.7]	544	52.6 [47.8-57.3]	321	36.9 [32.4-41.6]	68	9.7 [7.0-13.3]	938	100	7.9[a]	-6.6[a]	-1.3
Age																	

18-29	55	27.4 [20.6-35.5]	92	47.5 [39.1-55.9]	51	24.3 [17.9-32.1]	79	37.9 [30.2-46.3]	78	41.1 [33.0-49.6]	40	19.9 [14.0-27.6]	200	100	10.5[b]	-6.4	-4.4
30-44	159	32.8 [27.6-38.5]	241	44.6 [38.9-50.4]	105	21.6 [17.1-26.9]	195	37.9 [32.4-43.7]	194	39.4 [33.9-45.3]	113	21.3 [17.0-26.5]	511	100	5.1	-5.1	-0.3
45-59	309	36.9 [32.3-41.8]	295	42 [37.0-47.1]	128	19.8 [16.0-24.3]	337	39.6 [34.9-44.6]	260	37.7 [32.8-42.8]	132	20.2 [16.3-24.7]	738	100	2.7	-4.3	0.3
60+	824	54.9 [51.1-58.6]	458	33 [29.5-36.7]	128	11.3 [8.9-14.3]	860	55.7 [51.9-59.4]	424	32.6 [29.2-36.3]	125	11 [8.5-14.1]	1,421	100	0.8	-0.4	-0.3
Household gun ownership																	
No guns in home	845	35 [32.0-38.1]	790	42.8 [39.5-46.1]	341	21.5 [18.8-24.5]	950	39.6 [36.5-42.9]	685	38.6 [35.4-41.9]	331	20.2 [17.6-23.1]	1,989	100	4.7[a]	-4.2[b]	-1.3
Gun owner	338	57.5 [50.5-64.1]	163	35 [28.7-41.9]	24	7.1 [4.0-12.2]	346	56.6 [49.7-63.3]	148	33.2 [27.0-40.0]	34	10.1 [6.3-15.8]	529	100	-0.8	-1.9	3.1
Non-owner living with owner	97	36.9 [28.4-46.3]	93	42 [32.7-52.0]	29	21 [13.5-31.2]	109	50.1 [40.4-59.8]	79	33.9 [25.7-43.3]	28	15.8 [9.3-25.4]	219	100	13.2[b]	-8.1	-5.3
Acquired firearms due to the coronavirus pandemic																	
Non-gun owner	1009	35.8 [33.0-38.6]	923	42.3 [39.3-45.4]	388	20.8 [18.4-23.6]	1125	40.8 [37.9-43.8]	808	38.1 [35.2-41.2]	376	19.4 [17.0-22.0]	2,341	100	5.1[a]	-4.2[b]	-1.5
Gun owner, no purchase	332	57.3 [50.3-64.1]	159	35 [28.6-42.0]	24	7.3 [4.1-12.5]	341	56.9 [49.8-63.6]	143	32.7 [26.4-39.7]	34	10.4 [6.5-16.2]	519	100	-0.5	-2.3	3.1
Gun owner, purchase	6	63.3 [27.4-88.8]	4	36.7 [11.2-72.6]	0	0 [NA]	5	47.5 [16.6-80.4]	5	52.5 [19.6-83.4]	0	0 [NA]	10	100	-15.8	15.8	0.0

See footnotes at the end of eAppendix 18.

eAppendix 11. Percentage of Respondents Worried About Assault Happening to Them Before and During the COVID-19 Pandemic, 2020 California Safety and Well-being Survey

	Before the pandemic						During the pandemic						Total		During - Before		
	Not worried		Somewhat worried		Very worried		Not worried		Somewhat worried		Very worried				Not	Somewhat	Very
	n	% [95% CI]	n	% [95% CI]	n	% [95% CI]	n	% [95% CI]	n	% [95% CI]	n	% [95% CI]	n	%	PD	PD	PD
Total	1291	39.9 [37.3-42.6]	1264	45.8 [43.1-48.6]	293	13.3 [11.4-15.4]	1193	36.5 [33.9-39.2]	1222	43.9 [41.1-46.7]	433	18.6 [16.4-20.9]	2,870	100	-3.4[b]	-2.0	5.3[a]
Education																	
Less than high school	38	23.7 [17.0-32.1]	70	47.1 [38.4-56.0]	47	26.7 [19.6-35.2]	40	24.2 [17.5-32.4]	64	43.4 [34.8-52.4]	53	31.1 [23.5-39.9]	158	100	0.4	-3.7	4.4
High school	128	37.2 [30.8-44.1]	166	45.9 [39.2-52.8]	67	15.9 [11.7-21.4]	101	28.7 [22.8-35.4]	146	41.5 [35.0-48.4]	110	27.6 [22.0-34.0]	364	100	-8.6[b]	-4.4	11.6[a]
Some college	422	44 [39.6-48.5]	419	44.7 [40.3-49.2]	89	10.5 [8.0-13.7]	379	40.3 [35.9-44.8]	423	44.8 [40.4-49.3]	131	14.4 [11.5-17.8]	938	100	-3.8	0.1	3.8[b]
Bachelor's degree	397	45.7 [40.3-51.2]	318	43.1 [37.7-48.7]	57	10.6 [7.1-15.4]	362	39.8 [34.7-45.2]	324	46.4 [40.9-52.0]	85	13 [9.4-17.8]	775	100	-5.9	3.3	2.5
Advanced degree	306	44.9 [38.6-51.3]	291	50.3 [43.8-56.7]	33	4.3 [2.8-6.6]	311	48.7 [42.3-55.2]	265	42.4 [36.1-49.0]	54	8.1 [5.8-11.3]	635	100	3.8	-7.9[b]	3.8[a]
Gender																	
Male	753	44.7 [40.7-48.7]	615	42.6 [38.6-46.7]	127	11.6 [9.1-14.7]	664	39.2 [35.3-43.2]	625	42 [38.0-46.1]	204	17.5 [14.4-20.9]	1,506	100	-5.5[b]	-0.5	5.9[a]
Female	538	35.6 [32.1-39.2]	649	48.8 [45.0-52.6]	166	14.8 [12.1-17.9]	529	34.1 [30.7-37.7]	597	45.6 [41.8-49.4]	229	19.6 [16.6-22.9]	1,364	100	-1.5	-3.2	4.8[a]
Race/ethnicity																	
White, Non-Latinx	879	54.1 [50.3-57.8]	650	39.8 [36.2-43.5]	74	5.2 [3.6-7.3]	802	47.5 [43.7-51.2]	681	43.1 [39.4-46.8]	120	8.5 [6.5-10.9]	1,615	100	-6.6[a]	3.3	3.3[a]
Black, Non-Latinx	52	30.7 [21.3-42.0]	63	60.8 [49.0-71.5]	12	8.5 [3.8-18.0]	57	36.9 [26.4-48.7]	58	52.9 [41.0-64.5]	12	10.2 [4.8-20.4]	127	100	6.2	-7.9	1.7
Asian, Non-Latinx	70	34 [26.5-42.4]	111	52.9 [44.4-61.3]	27	13.1 [8.3-19.9]	73	34.5 [26.9-43.0]	99	49.7 [41.3-58.2]	36	15.8 [10.5-22.9]	208	100	0.5	-3.2	2.7
Other & 2+ races, Non-Latinx	40	46.6 [30.5-63.4]	24	40.8 [24.7-59.2]	6	9.9 [3.8-23.3]	40	49.1 [32.5-65.8]	24	39.5 [23.9-57.6]	7	11.4 [3.7-30.0]	71	100	2.5	-1.3	1.5
Latinx	250	26.2 [22.1-30.8]	416	48.1 [43.1-53.1]	174	24.2 [20.1-28.9]	221	22.9 [19.1-27.3]	360	41.3 [36.5-46.3]	258	34 [29.4-38.9]	849	100	-3.3	-6.8[b]	9.7[a]
Income																	
Less than $25,000	135	27.6 [21.8-34.4]	193	43.3 [36.4-50.5]	96	25.6 [19.8-32.5]	138	25.5 [20.1-31.9]	163	40.3 [33.4-47.5]	125	31.5 [25.2-38.5]	431	100	-2.1	-3.0	5.9
$25,000-$59,999	284	28 [23.8-32.6]	367	53.3 [47.9-58.7]	89	18.5 [14.3-23.6]	265	26.2 [22.1-30.7]	341	49.3 [43.9-54.8]	133	23.6 [19.2-28.8]	744	100	-1.8	-4.0	5.2
$60,000-$99,999	353	41.7 [36.3-47.3]	334	46.4 [40.8-52.0]	64	11.2 [8.0-15.5]	345	40.9 [35.5-46.5]	306	39.1 [33.9-44.7]	102	19.6 [15.3-24.8]	757	100	-0.8	-7.2[b]	8.3[a]
$100,000 or more	519	51.1 [46.4-55.8]	370	41.8 [37.2-46.6]	44	6.4 [4.1-9.6]	445	44.7 [40.1-49.4]	412	44.7 [40.0-49.4]	73	9.7 [7.0-13.3]	938	100	-6.4[b]	2.8	3.4

© 2021 Kravitz-Wirtz N et al. *JAMA Network Open.*

Age																	
18-29	67	33 [25.6-41.3]	95	49.2 [40.8-57.6]	36	17 [11.7-24.2]	72	34.7 [27.1-43.2]	80	43.7 [35.6-52.3]	46	20.7 [14.9-28.2]	200	100	1.8	-5.5	3.7
30-44	164	35.2 [29.8-41.0]	260	47.6 [41.8-53.4]	82	16.3 [12.3-21.3]	158	30.9 [25.8-36.5]	228	44.5 [38.8-50.3]	119	23.1 [18.6-28.4]	511	100	-4.3	-3.1	6.8[a]
45-59	299	37.4 [32.6-42.3]	339	48.4 [43.3-53.5]	91	12.4 [9.6-16.0]	261	33.2 [28.6-38.1]	333	46.1 [41.1-51.2]	138	19.7 [15.9-24.1]	738	100	-4.2	-2.3	7.2[a]
60+	761	51.7 [48.0-55.4]	570	39.4 [35.8-43.1]	84	8.5 [6.2-11.5]	702	47 [43.3-50.7]	581	41.1 [37.5-44.8]	130	11.2 [8.7-14.2]	1,421	100	-4.7[b]	1.7	2.7
Household gun ownership																	
No guns in home	845	36.7 [33.6-39.8]	897	47.9 [44.5-51.2]	234	14.6 [12.3-17.2]	805	34.6 [31.6-37.8]	841	44.8 [41.5-48.1]	331	19.7 [17.1-22.5]	1,989	100	-2.1	-3.1	5.1[a]
Gun owner	299	58.4 [51.8-64.8]	207	35.3 [29.4-41.6]	23	6.3 [3.4-11.4]	247	43.3 [36.8-50.1]	232	43.7 [37.2-50.4]	47	12.5 [8.3-18.5]	529	100	-15.1[a]	8.4[b]	6.3[b]
Non-owner living with owner	96	40.4 [31.4-50.2]	99	43.9 [34.6-53.7]	21	15.2 [8.8-25.0]	96	44.8 [35.4-54.7]	90	36.7 [28.0-46.3]	32	18.3 [11.5-27.8]	219	100	4.4	-7.3	3.1
Acquired firearms due to the coronavirus pandemic																	
Non-gun owner	992	36.6 [33.8-39.5]	1057	47.7 [44.7-50.8]	270	14.5 [12.4-16.9]	946	35.3 [32.5-38.2]	990	43.9 [40.9-47.0]	386	19.6 [17.2-22.2]	2,341	100	-1.3	-3.8[b]	5.1[a]
Gun owner, no purchase	294	58.5 [51.7-64.9]	203	35.3 [29.3-41.8]	22	6.2 [3.3-11.5]	244	43.2 [36.6-50.1]	227	43.8 [37.1-50.6]	45	12.6 [8.3-18.7]	519	100	-15.2[a]	8.5[b]	6.3[b]
Gun owner, purchase	5	58.3 [24.4-85.9]	4	34.4 [10.5-70.2]	1	7.2 [0.9-39.3]	3	47.2 [16.1-80.6]	5	41.1 [13.7-75.4]	2	11.7 [2.5-40.9]	10	100	-11.1	6.6	4.5

See footnotes at the end of eAppendix 18.

eAppendix 12. Percentage of Respondents Worried About Robbery Happening to Them Before and During the COVID-19 Pandemic, 2020 California Safety and Well-being Survey

	Before the pandemic						During the pandemic						Total		During - Before		
	Not worried		Somewhat worried		Very worried		Not worried		Somewhat worried		Very worried				Not	Somewhat	Very
	n	% [95% CI]	n	% [95% CI]	n	% [95% CI]	n	% [95% CI]	n	% [95% CI]	n	% [95% CI]	n	%	PD	PD	PD
Total	1048	33.5 [31.0-36.2]	1469	50.2 [47.4-52.9]	330	15.3 [13.3-17.6]	997	30.8 [28.3-33.4]	1364	46.3 [43.5-49.0]	487	22 [19.6-24.5]	2870	100	-2.7	-3.9[b]	6.6[a]
Education																	
Less than high school	30	19.3 [13.2-27.4]	67	44.9 [36.3-53.9]	58	34 [26.1-42.8]	27	18 [12.1-25.9]	68	42.8 [34.3-51.8]	62	37.9 [29.7-46.8]	158	100	-1.4	-2.1	3.9
High school	97	30 [24.0-36.9]	182	49.9 [43.1-56.7]	79	18.5 [14.0-24.1]	86	25.8 [20.1-32.5]	157	42.9 [36.3-49.7]	115	29.8 [24.0-36.3]	364	100	-4.2	-7.0	11.2[a]
Some college	362	40.4 [36.0-44.9]	473	48.4 [44.0-52.9]	96	10.3 [7.9-13.2]	335	35.6 [31.4-40.1]	445	46.3 [41.9-50.8]	150	17.3 [14.1-21.0]	938	100	-4.7	-2.1	7.0[a]
Bachelor's degree	318	36.5 [31.5-41.8]	389	51.1 [45.6-56.6]	66	11.8 [8.0-17.1]	298	34.2 [29.4-39.5]	378	47.8 [42.3-53.3]	97	17.4 [13.1-22.8]	775	100	-2.3	-3.3	5.6[b]
Advanced degree	241	34.4 [28.8-40.5]	358	59.1 [52.8-65.1]	31	6.1 [3.7-9.8]	251	36.8 [31.0-43.0]	316	53 [46.6-59.3]	63	9.5 [6.7-13.4]	635	100	2.3	-6.1	3.4
Gender																	
Male	624	38.7 [34.8-42.7]	723	46.9 [42.8-50.9]	148	13.4 [10.8-16.6]	580	34.8 [31.0-38.7]	691	45.1 [41.1-49.2]	223	18.9 [15.8-22.5]	1,506	100	-3.9	-1.7	5.5[a]
Female	424	28.8 [25.6-32.3]	746	53.2 [49.3-56.9]	182	17 [14.2-20.3]	417	27.2 [24.1-30.6]	673	47.3 [43.5-51.1]	264	24.7 [21.4-28.4]	1,364	100	-1.6	-5.9[a]	7.7[a]
Race/ethnicity																	
White, Non-Latinx	697	44.5 [40.8-48.2]	825	49.3 [45.6-53.1]	81	5.3 [3.9-7.2]	665	40.6 [37.0-44.3]	793	48.4 [44.7-52.2]	145	10 [7.9-12.5]	1,615	100	-3.9	-0.9	4.7[a]
Black, Non-Latinx	44	32.6 [22.3-44.8]	74	59.1 [46.8-70.3]	9	8.3 [3.5-18.4]	48	32.8 [22.6-45.0]	68	56.2 [44.1-67.7]	11	11 [5.3-21.3]	127	100	0.2	-2.9	2.6
Asian, Non-Latinx	52	23.1 [16.9-30.8]	118	59.7 [51.2-67.6]	36	16.4 [11.0-23.8]	53	23.3 [17.0-31.1]	104	54.1 [45.6-62.4]	51	22.5 [16.3-30.3]	208	100	0.2	-5.5	6.1
Other & 2+ races, Non-Latinx	37	51.9 [35.0-68.5]	29	40.1 [24.6-58.0]	5	7.9 [2.8-20.2]	36	45.4 [29.4-62.3]	25	31.3 [17.9-48.8]	10	23.3 [10.8-43.4]	71	100	-6.6	-8.9	15.4[b]
Latinx	218	23.1 [19.1-27.5]	423	46.6 [41.7-51.6]	199	28.8 [24.4-33.6]	195	20.4 [16.7-24.7]	374	40 [35.3-45.0]	270	37.9 [33.2-43.0]	849	100	-2.6	-6.6[b]	9.1[a]
Income																	
Less than $25,000	119	25.1 [19.4-31.7]	192	40.9 [34.1-48.0]	110	30.1 [23.8-37.1]	126	24.1 [18.7-30.6]	168	37.8 [31.1-45.0]	130	34.8 [28.3-41.9]	431	100	-0.9	-3.0	4.7
$25,000-$59,999	238	26 [21.7-30.7]	409	55.2 [49.7-60.5]	94	18.8 [14.6-23.7]	212	21.9 [18.0-26.4]	377	50.9 [45.4-56.3]	150	26.8 [22.1-32.1]	744	100	-4.0	-4.3	8.1[a]
$60,000-$99,999	291	33.8 [28.8-39.3]	384	50.3 [44.7-55.9]	79	15.4 [11.6-20.2]	281	32.3 [27.3-37.7]	358	44.5 [39.1-50.1]	115	23.1 [18.4-28.5]	757	100	-1.5	-5.8	7.6[a]
$100,000 or more	400	41.4 [36.8-46.0]	484	50.6 [45.9-55.3]	47	7.3 [4.9-10.8]	378	38.1 [33.7-42.7]	461	47.7 [43.0-52.4]	92	13.3 [10.1-17.3]	938	100	-3.3	-2.9	5.9[a]

© 2021 Kravitz-Wirtz N et al. *JAMA Network Open.*

Age																	
18-29	76	40.4 [32.4-48.9]	96	45.6 [37.4-54.0]	26	13.3 [8.6-20.0]	71	32.9 [25.6-41.1]	80	42.4 [34.3-51.0]	46	23.8 [17.4-31.8]	200	100	-7.5	-3.1	10.6[a]
30-44	131	26.8 [21.9-32.3]	273	51 [45.2-56.8]	102	21.2 [16.7-26.5]	124	26.1 [21.2-31.6]	236	44.1 [38.4-49.9]	145	28.6 [23.6-34.2]	511	100	-0.7	-6.9[b]	7.4[a]
45-59	256	31.1 [26.8-35.8]	369	51.4 [46.3-56.4]	109	16.4 [12.9-20.5]	233	28 [23.8-32.7]	356	48.2 [43.1-53.3]	144	22.8 [18.7-27.4]	738	100	-3.1	-3.2	6.4[a]
60+	585	39.1 [35.6-42.7]	731	50.8 [47.0-54.5]	93	9.1 [6.8-12.1]	569	37.5 [34.0-41.1]	692	49 [45.3-52.7]	152	12.8 [10.3-15.9]	1,421	100	-1.6	-1.8	3.8[b]
Household gun ownership																	
No guns in home	696	31.4 [28.4-34.5]	1013	50.4 [47.1-53.8]	267	17.4 [14.9-20.2]	672	28.9 [26.0-31.9]	939	46.9 [43.6-50.2]	365	23.4 [20.6-26.4]	1,989	100	-2.5	-3.5	6.0[a]
Gun owner	232	45.2 [38.5-52.1]	269	47.3 [40.6-54.0]	28	7.5 [4.5-12.1]	208	37.4 [31.0-44.2]	261	48.1 [41.4-54.8]	59	14.6 [10.1-20.5]	529	100	-7.9	0.8	7.1[a]
Non-owner living with owner	72	31.4 [23.4-40.6]	126	56.2 [46.5-65.5]	20	12.2 [6.8-21.0]	81	39.4 [30.3-49.2]	100	38.8 [30.0-48.3]	37	21.5 [13.9-31.7]	219	100	8.0	-17.4[a]	9.3
Acquired firearms due to the coronavirus pandemic																	
Non-gun owner	816	31.4 [28.7-34.3]	1200	50.7 [47.6-53.7]	302	16.7 [14.4-19.3]	789	29.6 [27.0-32.4]	1103	45.9 [42.9-49.0]	428	23.3 [20.7-26.1]	2,341	100	-1.8	-4.7[a]	6.6[a]
Gun owner, no purchase	228	45 [38.2-51.9]	263	47.3 [40.6-54.2]	28	7.7 [4.6-12.4]	205	37 [30.6-43.9]	256	48.4 [41.6-55.2]	57	14.6 [10.1-20.7]	519	100	-8.0	1.0	6.9[a]
Gun owner, purchase	4	55.6 [22.3-84.5]	6	44.4 [15.5-77.7]	0	0 [NA]	3	52.7 [20.2-83.1]	5	35.6 [11.2-70.8]	2	11.7 [2.5-40.9]	10	100	-2.9	-8.8	11.7

See footnotes at the end of eAppendix 18.

© 2021 Kravitz-Wirtz N et al. *JAMA Network Open.*

eAppendix 13. Percentage of Respondents Worried About Police Violence Happening to Them Before and During the COVID-19 Pandemic, 2020 California Safety and Well-being Survey

	Before the pandemic						During the pandemic						Total		During - Before		
	Not worried		Somewhat worried		Very worried		Not worried		Somewhat worried		Very worried				Not	Somewhat	Very
	n	% [95% CI]	n	% [95% CI]	n	% [95% CI]	n	% [95% CI]	n	% [95% CI]	n	% [95% CI]	n	%	PD	PD	PD
Total	1798	53.7 [50.8-56.5]	737	30.5 [28.0-33.3]	310	14.7 [12.7-17.0]	1673	48.1 [45.4-50.9]	739	30.9 [28.3-33.6]	434	19.7 [17.5-22.2]	2,870	100	-5.5[a]	0.3	5.0[a]
Education																	
Less than high school	56	33.4 [25.7-42.1]	56	38.4 [30.1-47.5]	41	25.6 [18.5-34.3]	49	28.6 [21.3-37.1]	58	39.8 [31.4-48.9]	48	29.6 [22.1-38.4]	158	100	-4.9	1.4	4.0
High school	184	49.4 [42.6-56.2]	107	31.2 [25.2-37.8]	65	17.7 [12.9-23.9]	156	39.5 [33.0-46.3]	104	32.2 [26.2-39.0]	97	26 [20.4-32.5]	364	100	-9.9[a]	1.1	8.3[a]
Some college	593	58.1 [53.6-62.6]	242	28.2 [24.2-32.6]	99	13 [10.0-16.6]	577	55.7 [51.1-60.2]	232	27.5 [23.5-31.8]	122	16.1 [12.9-19.9]	938	100	-2.4	-0.8	3.1
Bachelor's degree	519	58.1 [52.4-63.6]	183	30 [24.9-35.6]	71	11.4 [8.0-16.0]	477	52.4 [46.9-58.0]	199	28.7 [23.9-34.0]	98	18.4 [14.0-23.7]	775	100	-5.7	-1.3	7.0[a]
Advanced degree	446	66.2 [59.6-72.2]	149	27.1 [21.4-33.6]	34	6.4 [3.8-10.5]	414	59.4 [52.7-65.8]	146	29.7 [23.5-36.8]	69	9.9 [7.2-13.4]	635	100	-6.8	2.6	3.5
Gender																	
Male	940	51.4 [47.3-55.5]	390	31.2 [27.4-35.2]	165	16.3 [13.2-20.0]	898	47.8 [43.8-51.8]	375	30.7 [26.9-34.7]	219	20.2 [16.9-24.0]	1,506	100	-3.6	-0.5	3.9
Female	858	55.7 [51.8-59.5]	347	29.9 [26.5-33.7]	145	13.2 [10.7-16.2]	775	48.5 [44.7-52.3]	364	31 [27.5-34.8]	215	19.3 [16.4-22.6]	1,364	100	-7.2[a]	1.1	6.1[a]
Race/ethnicity																	
White, Non-Latinx	1227	72.6 [68.9-76.0]	304	20.6 [17.6-24.0]	75	6 [4.3-8.4]	1166	67.4 [63.7-71.0]	316	21.3 [18.3-24.7]	124	10.4 [8.2-13.1]	1,615	100	-5.1[a]	0.7	4.3[a]
Black, Non-Latinx	40	25.8 [17.1-36.9]	46	37.9 [27.1-49.9]	41	36.4 [25.3-49.1]	40	28.8 [19.4-40.3]	49	35.4 [25.1-47.3]	38	35.8 [24.7-48.7]	127	100	3.0	-2.5	-0.5
Asian, Non-Latinx	107	50.6 [42.1-59.0]	77	37.2 [29.3-45.9]	24	12.2 [7.5-19.2]	99	46.1 [37.8-54.6]	74	37.5 [29.5-46.3]	34	16.1 [10.8-23.3]	208	100	-4.5	0.3	3.9
Other & 2+ races, Non-Latinx	48	73.1 [58.2-84.1]	16	19 [10.0-33.1]	6	5.6 [2.3-12.9]	47	58 [40.2-74.0]	16	35.3 [20.0-54.3]	8	6.7 [3.0-14.3]	71	100	-15.0	16.2	1.1
Latinx	376	34.9 [30.5-39.6]	294	39.6 [34.8-44.6]	164	23.5 [19.3-28.2]	321	28 [24.0-32.4]	284	38.5 [33.7-43.5]	230	31.1 [26.6-36.0]	849	100	-6.9[a]	-1.1	7.6[a]
Income																	
Less than $25,000	196	35.2 [29.0-41.9]	129	36.6 [29.8-43.9]	95	24 [18.2-31.0]	188	34.1 [28.1-40.8]	125	34.9 [28.2-42.1]	110	27.7 [21.6-34.7]	431	100	-1.1	-1.7	3.6
$25,000-$59,999	435	47.8 [42.4-53.2]	215	33.3 [28.4-38.7]	90	18.7 [14.4-23.9]	400	40 [35.0-45.2]	209	33.8 [28.7-39.4]	127	24.7 [20.1-30.0]	744	100	-7.8[a]	0.5	6.0[b]
$60,000-$99,999	496	52.8 [47.1-58.4]	189	34 [28.6-39.9]	68	12.5 [9.0-17.0]	452	46.2 [40.7-51.7]	205	34.3 [28.9-40.1]	99	19.5 [15.2-24.7]	757	100	-6.7[b]	0.3	7.0[a]
$100,000 or more	671	65 [60.2-69.5]	204	24.4 [20.5-28.8]	57	9.9 [7.0-13.7]	633	59.8 [55.0-64.5]	200	25.5 [21.4-30.0]	98	13.7 [10.5-17.6]	938	100	-5.2	1.1	3.8
Age																	

© 2021 Kravitz-Wirtz N et al. *JAMA Network Open.*

18-29	83	39.9 [32.0-48.4]	78	41 [33.0-49.6]	36	17.8 [12.2-25.2]	69	34 [26.5-42.4]	72	38.3 [30.3-46.9]	57	26.9 [20.3-34.9]	200	100	-5.9	-2.8	9.1[b]
30-44	248	46.8 [41.1-52.7]	171	33.7 [28.5-39.4]	83	18 [13.7-23.3]	207	37.9 [32.5-43.6]	177	36.3 [30.9-42.1]	120	23.9 [19.2-29.3]	511	100	-8.9[a]	2.6	5.9[b]
45-59	437	53.1 [48.0-58.2]	193	29.5 [25.0-34.5]	103	16.1 [12.5-20.5]	407	49.6 [44.6-54.7]	181	27.6 [23.1-32.5]	142	21.4 [17.5-26.0]	738	100	-3.5	-1.9	5.3[b]
60+	1030	69.8 [66.1-73.2]	295	21.9 [18.9-25.2]	88	7.9 [5.8-10.5]	990	66.2 [62.5-69.8]	309	23.8 [20.6-27.2]	115	9.2 [7.1-11.9]	1,421	100	-3.6	1.9	1.4
Household gun ownership																	
No guns in home	1162	48.7 [45.4-52.0]	560	34.3 [31.1-37.6]	248	15.9 [13.4-18.6]	1079	43.8 [40.6-47.1]	555	33.7 [30.5-37.1]	555	21.4 [18.7-24.3]	1,989	100	-4.9[a]	-0.6	5.5[a]
Gun owner	412	73.1 [66.3-79.0]	92	18.3 [13.7-24.0]	25	8.6 [4.8-15.0]	390	65.6 [58.5-72.0]	91	20.3 [15.3-26.5]	91	13 [8.4-19.6]	529	100	-7.5	2.0	4.4
Non-owner living with owner	150	66.4 [56.6-75.0]	53	19.7 [13.9-27.2]	16	13.9 [7.5-24.3]	129	54.5 [44.6-64.1]	61	27.6 [19.7-37.3]	61	17.9 [11.1-27.5]	219	100	-11.9[b]	7.9	4.0
Acquired firearms due to the coronavirus pandemic																	
Non-gun owner	1386	50.2 [47.1-53.2]	645	32.7 [29.9-35.8]	285	15.8 [13.5-18.3]	1283	45 [42.0-48.0]	648	32.8 [29.9-35.8]	391	20.9 [18.5-23.6]	2,341	100	-5.2[a]	0.0	5.1[a]
Gun owner, no purchase	404	72.7 [65.7-78.7]	90	18.5 [13.8-24.3]	25	8.8 [4.9-15.3]	382	64.9 [57.8-71.5]	91	20.8 [15.7-27.1]	41	13.1 [8.4-19.9]	519	100	-7.7	2.3	4.3
Gun owner, purchase	8	90.5 [65.6-98.0]	2	9.5 [2.0-34.4]	0	0 [NA]	8	90.5 [65.6-98.0]	0	0 [NA]	2	9.5 [2.0-34.4]	10	100	0.0	-9.5	9.5

See footnotes at the end of eAppendix 18.

© 2021 Kravitz-Wirtz N et al. *JAMA Network Open.*

eAppendix 14. Percentage of Respondents Worried About an Unintentional Shooting Happening to Them Before and During the COVID-19 Pandemic, 2020 California Safety and Well-being Survey

	Before the pandemic						During the pandemic						Total		During - Before		
	Not worried		Somewhat worried		Very worried		Not worried		Somewhat worried		Very worried				Not	Somewhat	Very
	n	% [95% CI]	n	% [95% CI]	n	% [95% CI]	n	% [95% CI]	n	% [95% CI]	n	% [95% CI]	n	%	PD	PD	PD
Total	1837	56.2 [53.3-59.0]	795	32.2 [29.6-34.9]	216	10.5 [8.7-12.5]	1697	51 [48.2-53.8]	853	33.9 [31.2-36.6]	298	14.2 [12.2-16.4]	2,870	100	-5.2[a]	1.7	3.7[a]
Education																	
Less than high school	51	33 [25.2-41.8]	62	40.9 [32.5-49.8]	41	23.6 [16.8-32.0]	46	27.5 [20.3-36.0]	59	40.8 [32.3-49.9]	50	30 [22.5-38.7]	158	100	-5.5	-0.1	6.5
High school	186	50.8 [44.0-57.6]	116	35.9 [29.5-42.9]	56	11.2 [7.8-15.8]	158	42.7 [36.1-49.5]	128	38.4 [31.9-45.3]	72	17.6 [13.1-23.3]	364	100	-8.1[b]	2.5	6.4[b]
Some college	627	65 [60.6-69.2]	250	26.7 [23.0-30.9]	54	7.5 [5.2-10.6]	570	58.9 [54.5-63.3]	275	29 [25.2-33.2]	87	11.3 [8.6-14.6]	938	100	-6.1[b]	2.3	3.8[b]
Bachelor's degree	518	58.5 [52.7-64.0]	210	32.7 [27.5-38.4]	45	8.3 [5.3-12.9]	490	55.3 [49.7-60.9]	224	34 [28.8-39.7]	59	10.1 [6.9-14.7]	775	100	-3.2	1.3	1.8
Advanced degree	455	66 [59.3-72.2]	157	29.1 [23.2-35.9]	20	4.6 [2.5-8.4]	433	65 [58.3-71.2]	167	30.8 [24.7-37.6]	30	3.5 [2.2-5.6]	635	100	-1.0	1.7	-1.1
Gender																	
Male	1013	58.4 [54.2-62.4]	383	31 [27.2-35.1]	97	9.2 [6.9-12.2]	938	54.5 [50.4-58.6]	415	31.2 [27.4-35.2]	143	13.3 [10.6-16.6]	1,506	100	-3.9	0.1	4.1[a]
Female	824	54.2 [50.3-58.0]	412	33.2 [29.7-36.9]	119	11.6 [9.2-14.6]	759	47.8 [44.0-51.5]	438	36.3 [32.7-40.1]	155	14.9 [12.3-18.1]	1,364	100	-6.4[a]	3.1	3.3
Race/ethnicity																	
White, Non-Latinx	1236	76.8 [73.5-79.7]	333	19.7 [16.9-22.7]	40	2.8 [1.8-4.5]	1138	67.6 [64.0-71.1]	404	25.8 [22.7-29.2]	64	5.6 [3.9-8.1]	1,615	100	-9.1[a]	6.1[a]	2.8[a]
Black, Non-Latinx	69	46.9 [35.3-58.8]	47	42.3 [30.9-54.6]	11	10.9 [5.0-22.2]	70	46 [34.5-58.0]	43	42.3 [30.8-54.7]	14	11.7 [5.9-21.9]	127	100	-0.9	0.0	0.9
Asian, Non-Latinx	96	42.8 [34.8-51.3]	90	46.8 [38.4-55.4]	21	10 [6.0-16.4]	96	44.9 [36.7-53.4]	82	42.5 [34.2-51.2]	30	12.6 [8.1-19.0]	208	100	2.0	-4.3	2.6
Other & 2+ races, Non-Latinx	46	60.3 [42.8-75.5]	19	33.5 [19.2-51.6]	6	6.2 [2.5-14.8]	45	61.8 [44.2-76.7]	20	31.6 [17.8-49.7]	6	6.6 [2.2-18.1]	71	100	1.5	-1.9	0.4
Latinx	390	38 [33.4-42.8]	306	39.4 [34.6-44.4]	138	20.2 [16.3-24.8]	348	33.2 [28.9-37.9]	304	38.9 [34.1-43.9]	184	26.3 [22.0-31.0]	849	100	-4.8	-0.5	6.0[b]
Income																	
Less than $25,000	203	38 [31.6-44.9]	135	37.3 [30.5-44.6]	83	21 [15.7-27.7]	190	33.7 [27.7-40.4]	137	37.8 [30.9-45.1]	95	25 [19.2-31.9]	431	100	-4.3	0.5	4.0
$25,000-$59,999	442	48.2 [42.9-53.7]	233	37.7 [32.5-43.2]	64	13.2 [9.7-17.7]	395	40.9 [35.9-46.1]	251	41.7 [36.3-47.3]	95	17.3 [13.6-21.8]	744	100	-7.4[b]	4.0	4.1
$60,000-$99,999	495	56.4 [50.7-62.0]	215	33.1 [27.9-38.7]	44	9.6 [6.4-14.0]	474	53.3 [47.6-58.9]	210	30.6 [25.7-36.0]	69	15.7 [11.6-21.1]	757	100	-3.2	-2.5	6.2[a]
$100,000 or more	697	68 [63.2-72.5]	212	26.2 [22.1-30.8]	25	5.2 [3.1-8.5]	638	62.6 [57.8-67.2]	255	29.4 [25.2-34.0]	39	7.1 [4.6-10.6]	938	100	-5.4	3.2	1.8
Age																	

© 2021 Kravitz-Wirtz N et al. *JAMA Network Open.*

18-29	109	53.7 [45.2-61.9]	62	32.5 [25.0-41.0]	26	12 [7.7-18.2]	102	49.9 [41.5-58.3]	63	33.4 [25.9-41.8]	33	15.9 [10.7-23.2]	200	100	-3.8	0.9	3.9
30-44	248	47 [41.3-52.8]	183	36.1 [30.7-41.9]	72	15.7 [11.7-20.7]	222	40.6 [35.1-46.3]	194	41.6 [35.9-47.5]	88	16.6 [12.7-21.4]	511	100	-6.4	5.5	0.9
45-59	447	54.7 [49.5-59.7]	223	35.1 [30.3-40.2]	62	8.6 [6.1-12.1]	412	48.6 [43.5-53.6]	225	33.9 [29.2-38.9]	96	16.5 [12.7-21.0]	738	100	-6.1[b]	-1.2	7.8[a]
60+	1033	69.1 [65.3-72.6]	327	24.9 [21.7-28.4]	56	5.7 [3.9-8.2]	961	65.3 [61.6-68.8]	371	25.8 [22.7-29.2]	81	8.3 [6.1-11.1]	1,421	100	-3.8	0.9	2.6
Household gun ownership																	
No guns in home	1196	51.6 [48.3-55.0]	601	35.5 [32.3-38.8]	180	11.8 [9.7-14.2]	1113	47.6 [44.3-50.9]	612	35.2 [32.0-38.5]	252	16.5 [14.1-19.3]	1,989	100	-4.1[b]	-0.3	4.7[a]
Gun owner	417	75.5 [68.8-81.2]	96	19.7 [14.7-25.9]	14	4.4 [1.9-10.0]	386	65.7 [58.6-72.1]	119	26.3 [20.6-33.0]	23	8 [4.4-14.2]	529	100	-9.9[a]	6.7	3.5
Non-owner living with owner	144	60.7 [50.6-69.9]	64	29 [21.1-38.6]	11	10.3 [5.0-20.0]	128	57.7 [47.8-67.0]	76	29.6 [21.7-38.8]	12	10.9 [5.6-20.2]	219	100	-3.0	0.5	0.7
Acquired firearms due to the coronavirus pandemic																	
Non-gun owner	1420	52.7 [49.6-55.8]	699	34.4 [31.5-37.4]	202	11.6 [9.6-13.8]	1311	48.4 [45.3-51.4]	734	35.2 [32.3-38.3]	275	15.3 [13.1-17.7]	2,341	100	-4.3[a]	0.8	3.7[a]
Gun owner, no purchase	408	75 [68.2-80.9]	95	20 [14.9-26.4]	14	4.6 [2.0-10.2]	380	65.7 [58.4-72.2]	115	26.1 [20.3-32.9]	23	8.2 [4.5-14.5]	519	100	-9.4[b]	6.1	3.6
Gun owner, purchase	9	95.5 [72.0-99.4]	1	4.5 [0.6-28.0]	0	0 [NA]	6	65.6 [29.8-89.5]	4	34.4 [10.5-70.2]	0	0 [NA]	10	100	-29.9[b]	29.9[b]	0.0

See footnotes at the end of eAppendix 18.

eAppendix 15. Percentage of Respondents Worried About Being Hit by a Stray Bullet Before and During the COVID-19 Pandemic, 2020 California Safety and Well-being Survey

	Before the pandemic						During the pandemic						Total		During - Before		
	Not worried		Somewhat worried		Very worried		Not worried		Somewhat worried		Very worried				Not	Somewhat	Very
	n	% [95% CI]	n	% [95% CI]	n	% [95% CI]	n	% [95% CI]	n	% [95% CI]	n	% [95% CI]	n	%	PD	PD	PD
Total	1820	54.7 [51.9-57.5]	799	33.7 [31.0-36.5]	229	10.8 [9.1-12.7]	1642	48.8 [46.0-51.6]	898	35.8 [33.1-38.5]	303	14.3 [12.3-16.5]	2,870	100	-5.9[a]	2.0	3.5[a]
Education																	
Less than high school	37	21.4 [15.1-29.4]	73	51.3 [42.5-60.1]	47	25.9 [19.0-34.4]	37	22 [15.7-30.1]	64	42.7 [34.2-51.7]	53	32.3 [24.5-41.2]	158	100	0.7	-8.6	6.3
High school	174	46.9 [40.2-53.8]	123	39.9 [33.3-46.9]	60	11.7 [8.4-15.9]	143	39.2 [32.7-46.0]	136	40.5 [34.0-47.4]	78	18.8 [14.1-24.5]	364	100	-7.7	0.6	7.1[a]
Some college	602	62.1 [57.7-66.4]	266	28.8 [24.9-33.0]	66	8.5 [6.2-11.6]	531	54.7 [50.2-59.1]	318	34.2 [30.2-38.5]	84	10.5 [7.9-13.9]	938	100	-7.4[a]	5.4[b]	2.0
Bachelor's degree	553	66.9 [61.3-72.1]	185	25.6 [21.0-30.8]	36	7 [4.1-11.5]	501	58.2 [52.5-63.7]	216	33.4 [28.3-39.0]	53	7.7 [4.9-12.0]	775	100	-8.7[a]	7.8[a]	0.8
Advanced degree	454	70.1 [63.5-75.9]	152	26.8 [21.1-33.5]	20	2.7 [1.5-4.6]	430	66.7 [60.2-72.7]	164	27.7 [22.0-34.3]	35	4.7 [2.9-7.5]	635	100	-3.3	0.9	2.1
Gender																	
Male	1003	56.5 [52.3-60.6]	384	32.6 [28.7-36.7]	108	10.1 [7.7-13.1]	914	51 [46.9-55.1]	430	34.6 [30.7-38.7]	147	13.3 [10.6-16.6]	1,506	100	-5.5[b]	2.0	3.2
Female	817	53 [49.2-56.9]	415	34.8 [31.2-38.6]	121	11.4 [9.1-14.2]	728	46.8 [43.0-50.6]	468	36.8 [33.3-40.6]	156	15.1 [12.3-18.4]	1,364	100	-6.3[a]	2.1	3.8[b]
Race/ethnicity																	
White, Non-Latinx	1238	75.5 [72.1-78.7]	326	20.8 [17.9-24.1]	38	2.8 [1.7-4.5]	1125	67.4 [63.8-70.8]	411	27.6 [24.3-31.1]	70	4.3 [3.0-6.1]	1,615	100	-8.1[a]	6.8[a]	1.5
Black, Non-Latinx	67	41.9 [31.0-53.7]	47	47.7 [35.9-59.8]	13	10.4 [5.0-20.2]	61	37.9 [27.4-49.5]	54	52.9 [41.0-64.5]	11	9.1 [4.1-18.9]	127	100	-4.1	5.2	-1.3
Asian, Non-Latinx	115	52.3 [43.7-60.7]	72	38.2 [30.2-46.9]	21	9.5 [5.6-15.7]	98	46.7 [38.3-55.2]	85	41.5 [33.4-50.1]	24	11.7 [7.1-18.5]	208	100	-5.6	3.3	2.1
Other & 2+ races, Non-Latinx	47	65.5 [48.6-79.3]	21	30.9 [17.9-47.8]	3	3.6 [1.0-12.5]	45	54.1 [36.9-70.5]	20	27.5 [15.4-44.1]	6	18.4 [7.2-39.7]	71	100	-11.4	-3.4	14.8[b]
Latinx	353	31.7 [27.5-36.2]	333	45.4 [40.5-50.5]	154	21.6 [17.7-26.1]	313	28.5 [24.4-32.9]	328	41.2 [36.4-46.2]	192	27.9 [23.5-32.7]	849	100	-3.2	-4.2	6.2[b]
Income																	
Less than $25,000	182	34 [27.9-40.8]	154	41.7 [34.7-49.0]	87	21.3 [16.1-27.6]	176	33.9 [27.7-40.8]	149	38.9 [32.1-46.1]	96	23.9 [18.2-30.6]	431	100	-0.1	-2.8	2.6
$25,000-$59,999	436	45.9 [40.7-51.3]	232	38.8 [33.4-44.4]	73	15.2 [11.5-19.9]	373	36.3 [31.5-41.3]	270	43.4 [38.0-48.9]	95	19.8 [15.4-25.1]	744	100	-9.7[a]	4.6	4.6
$60,000-$99,999	486	49.7 [44.1-55.3]	219	40.6 [35.0-46.5]	46	9.2 [6.3-13.2]	446	46.1 [40.7-51.7]	233	38.6 [33.1-44.3]	71	14.1 [10.3-18.8]	757	100	-3.6	-2.0	4.9[b]
$100,000 or more	716	71.1 [66.4-75.4]	194	23.5 [19.6-27.9]	23	4.8 [2.8-8.1]	647	63.9 [59.2-68.4]	246	28.2 [24.1-32.7]	41	7.2 [4.7-10.7]	938	100	-7.2[a]	4.7	2.3
Age																	

© 2021 Kravitz-Wirtz N et al. *JAMA Network Open.*

18-29	109	53.8 [45.4-62.1]	65	35.1 [27.4-43.6]	24	10.3 [6.5-16.0]	104	50 [41.6-58.4]	64	33.2 [25.8-41.6]	30	16 [10.5-23.4]	200	100	-3.8	-1.9	5.6
30-44	244	46.3 [40.5-52.1]	191	38.4 [32.9-44.2]	72	14.5 [10.8-19.3]	209	39.9 [34.3-45.7]	197	40.1 [34.6-46.0]	98	18.7 [14.5-23.7]	511	100	-6.4	1.8	4.2
45-59	445	51.5 [46.3-56.5]	214	35.8 [30.8-41.0]	72	11.5 [8.5-15.4]	391	45.1 [40.2-50.1]	245	37.5 [32.6-42.6]	91	15.2 [11.6-19.6]	738	100	-6.3[b]	1.7	3.7
60+	1022	67.5 [63.7-71.2]	329	25.9 [22.5-29.6]	61	6.2 [4.4-8.8]	938	61.3 [57.5-65.0]	392	30.9 [27.4-34.6]	84	7.6 [5.6-10.3]	1,421	100	-6.2[a]	5.0[b]	1.3
Household gun ownership																	
No guns in home	1184	49.9 [46.6-53.2]	600	37 [33.7-40.3]	191	12.4 [10.3-14.9]	1080	45.2 [41.9-48.5]	642	37.1 [33.9-40.4]	249	16.5 [14.0-19.3]	1,989	100	-4.7[a]	0.2	4.1[a]
Gun owner	409	72.1 [65.2-78.0]	107	23.6 [18.2-30.1]	13	4.4 [1.9-9.9]	362	62.9 [55.9-69.3]	144	31.5 [25.4-38.3]	22	5.6 [3.0-10.3]	529	100	-9.2[b]	7.9[b]	1.2
Non-owner living with owner	143	62.3 [52.3-71.3]	62	28 [20.1-37.4]	12	9.4 [4.6-18.5]	130	55.2 [45.2-64.7]	68	28.8 [21.0-38.0]	20	15.8 [8.8-26.7]	219	100	-7.1	0.8	6.4
Acquired firearms due to the coronavirus pandemic																	
Non-gun owner	1411	51.6 [48.5-54.6]	692	35.5 [32.6-38.6]	216	11.9 [10.0-14.1]	1280	46.3 [43.3-49.3]	754	36.5 [33.6-39.6]	281	15.8 [13.6-18.4]	2,341	100	-5.3[a]	1.0	3.9[a]
Gun owner, no purchase	402	71.9 [64.8-78.0]	104	23.7 [18.2-30.3]	13	4.5 [1.9-10.1]	356	62.8 [55.8-69.4]	140	31.4 [25.2-38.4]	22	5.7 [3.0-10.5]	519	100	-9.0[b]	7.8[b]	1.3
Gun owner, purchase	7	80.1 [47.0-94.8]	3	19.9 [5.2-53.0]	0	0 [NA]	6	65.6 [29.8-89.5]	4	34.4 [10.5-70.2]	0	0 [NA]	10	100	-14.5	14.5	0.0

See footnotes at the end of eAppendix 18.

© 2021 Kravitz-Wirtz N et al. *JAMA Network Open.*

eAppendix 16. Percentage of Respondents Worried About a Violent Event Happening to Them in Their Home Before and During the COVID-19 Pandemic, 2020 California Safety and Well-being Survey

	Before the pandemic						During the pandemic						Total		During - Before		
	Not worried		Somewhat worried		Very worried		Not worried		Somewhat worried		Very worried				Not	Somewhat	Very
	n	% [95% CI]	n	% [95% CI]	n	% [95% CI]	n	% [95% CI]	n	% [95% CI]	n	% [95% CI]	n	%	PD	PD	PD
Total	2153	70.6 [67.9-73.1]	583	22.5 [20.2-24.9]	101	5.4 [4.1-7.1]	2085	69.4 [66.7-72.0]	615	21.9 [19.7-24.3]	138	7.2 [5.7-8.9]	2,870	100	-1.2	-0.6	1.7
Education																	
Less than high school	86	54 [45.0-62.7]	41	27.5 [20.3-36.1]	26	15.7 [10.1-23.6]	97	60.7 [51.7-69.1]	31	19.3 [13.3-27.2]	26	17.7 [11.7-25.8]	158	100	6.7	-8.2	2.0
High school	253	69 [62.2-75.0]	81	23 [17.7-29.3]	23	6 [3.5-10.2]	231	62.9 [56.1-69.3]	86	25.4 [19.9-31.9]	36	8.7 [5.6-13.4]	364	100	-6.0	2.5	2.7
Some college	688	71.3 [67.0-75.2]	210	24.3 [20.6-28.5]	32	3.6 [2.3-5.7]	676	70.7 [66.4-74.7]	214	23.9 [20.2-28.1]	41	4.5 [2.9-6.8]	938	100	-0.6	-0.4	0.8
Bachelor's degree	615	77.8 [72.8-82.0]	137	18.1 [14.4-22.4]	17	2.8 [1.2-6.5]	582	74.9 [69.8-79.3]	164	19.3 [15.6-23.8]	26	5.2 [2.9-9.3]	775	100	-2.9	1.3	2.5
Advanced degree	511	80.5 [75.1-84.9]	114	17.4 [13.2-22.7]	3	0.6 [0.2-2.5]	499	78.7 [73.1-83.3]	120	18.2 [13.8-23.7]	9	1.9 [0.9-4.1]	635	100	-1.8	0.8	1.3
Gender																	
Male	1169	71.8 [67.7-75.5]	276	21.8 [18.5-25.5]	43	4.8 [3.1-7.4]	1115	69.7 [65.7-73.4]	308	21.2 [18.0-24.8]	68	7.5 [5.4-10.5]	1,506	100	-2.0	-0.6	2.7
Female	984	69.5 [65.8-72.9]	307	23.1 [20.1-26.4]	58	6 [4.3-8.3]	970	69.1 [65.5-72.6]	307	22.5 [19.5-25.9]	70	6.8 [5.0-9.2]	1,364	100	-0.4	-0.6	0.8
Race/ethnicity																	
White, Non-Latinx	1305	77.8 [74.3-80.8]	280	19 [16.1-22.1]	22	2.5 [1.4-4.5]	1231	72.5 [68.9-75.9]	339	23 [19.9-26.4]	34	3.5 [2.2-5.6]	1,615	100	-5.3[a]	4.0[b]	1.0
Black, Non-Latinx	97	72.6 [60.5-82.1]	26	25.1 [15.9-37.4]	1	0.8 [0.1-5.7]	99	77.7 [66.9-85.7]	25	20.5 [12.8-31.3]	1	0.6 [0.1-4.2]	127	100	5.1	-4.6	-0.2
Asian, Non-Latinx	146	69.8 [61.7-76.9]	56	25.9 [19.4-33.7]	5	3.4 [1.3-9.0]	146	71.2 [63.0-78.2]	52	23.8 [17.4-31.8]	10	5 [2.4-10.0]	208	100	1.3	-2.0	1.5
Other & 2+ races, Non-Latinx	55	84 [70.9-91.9]	14	14.4 [7.0-27.4]	0	0 [NA]	54	72.8 [53.8-86.0]	16	27 [13.8-46.1]	1	0.2 [0.0-1.2]	71	100	-11.2	12.7	0.2
Latinx	550	60.6 [55.6-65.4]	207	25.6 [21.5-30.3]	73	11 [8.1-14.9]	555	63.3 [58.3-68.0]	183	19.5 [16.0-23.7]	92	14.2 [10.8-18.4]	849	100	2.6	-6.1[a]	3.2
Income																	
Less than $25,000	274	57.7 [50.4-64.6]	109	27 [21.3-33.6]	35	10.9 [6.8-17.2]	272	58.7 [51.5-65.6]	103	23.3 [18.2-29.3]	44	13.8 [9.0-20.4]	431	100	1.1	-3.7	2.9
$25,000-$59,999	532	64.7 [59.2-69.9]	166	25.3 [20.7-30.5]	39	8.8 [5.9-12.8]	525	65.6 [60.2-70.7]	164	23.2 [18.8-28.2]	46	9.4 [6.5-13.3]	744	100	0.9	-2.1	0.6
$60,000-$99,999	586	73.4 [68.1-78.2]	153	23.2 [18.7-28.4]	15	2.8 [1.3-5.6]	561	73.1 [67.9-77.8]	169	22.3 [18.0-27.2]	23	4.4 [2.5-7.5]	757	100	-0.3	-0.9	1.6

© 2021 Kravitz-Wirtz N et al. *JAMA Network Open.*

$100,000 or more	761	77.6 [73.2-81.4]	155	18.5 [15.1-22.6]	12	2.7 [1.3-5.6]	727	73.8 [69.1-77.9]	179	20.4 [16.7-24.6]	25	4.8 [2.9-8.0]	938	100	-3.8	1.8	2.1
Age																	
18-29	142	72.4 [64.5-79.1]	46	21 [15.1-28.5]	9	5.1 [2.5-10.1]	140	69 [60.7-76.2]	46	24 [17.5-32.0]	11	5.6 [2.8-10.9]	200	100	-3.4	3.0	0.5
30-44	337	64.5 [58.7-69.9]	134	26.4 [21.6-31.8]	29	6.8 [4.2-10.9]	324	63 [57.2-68.5]	132	23.9 [19.4-29.1]	44	10.9 [7.6-15.5]	511	100	-1.5	-2.5	4.1
45-59	537	69.4 [64.3-74.0]	161	22.7 [18.7-27.3]	32	6.7 [4.3-10.3]	521	70.8 [65.9-75.2]	163	20.4 [16.6-24.7]	48	7.7 [5.3-11.0]	738	100	1.4	-2.4	1.0
60+	1137	77.2 [73.6-80.5]	242	18.9 [16.0-22.2]	31	2.8 [1.5-5.2]	1100	75.3 [71.7-78.6]	274	20 [17.1-23.3]	35	3.4 [2.0-5.9]	1,421	100	-1.9	1.1	0.6
Household gun ownership																	
No guns in home	1481	69.7 [66.4-72.7]	401	22.9 [20.2-25.9]	84	5.8 [4.3-7.8]	1448	69.2 [66.0-72.3]	411	21.8 [19.2-24.7]	108	7.4 [5.7-9.4]	1,989	100	-0.4	-1.1	1.5
Gun owner	429	78 [71.5-83.4]	94	19.1 [14.2-25.2]	3	2.4 [0.7-8.1]	404	73.5 [66.7-79.4]	114	20.4 [15.6-26.2]	9	5.8 [2.6-12.5]	529	100	-4.5	1.3	3.4
Non-owner living with owner	158	71.2 [61.7-79.2]	51	21.6 [15.0-30.0]	10	7.2 [3.0-16.2]	153	69 [59.1-77.4]	52	22.9 [15.8-32.0]	13	8 [3.7-16.7]	219	100	-2.3	1.4	0.8
Acquired firearms due to the coronavirus pandemic																	
Non-gun owner	1724	69.2 [66.3-72.0]	489	23.1 [20.6-25.8]	98	6 [4.5-7.8]	1681	68.7 [65.7-71.5]	501	22.2 [19.7-24.8]	129	7.4 [5.8-9.3]	2,341	100	-0.6	-0.9	1.4
Gun owner, no purchase	419	77.5 [70.8-83.0]	94	19.6 [14.6-25.8]	3	2.5 [0.7-8.3]	397	73.2 [66.3-79.2]	111	20.5 [15.6-26.5]	9	6 [2.7-12.8]	519	100	-4.3	0.9	3.5
Gun owner, purchase	10	100 [NA]	0	0 [NA]	0	0 [NA]	7	84.6 [56.0-96.0]	3	15.4 [4.0-44.0]	0	0 [NA]	10	100	-15.4	15.4	0.0

See footnotes at the end of eAppendix 18.

© 2021 Kravitz-Wirtz N et al. *JAMA Network Open.*

eAppendix 17. Percentage of respondents Worried About a Violent Event Happening to Them in Their Neighborhood Before and During the COVID-19 Pandemic, 2020 California Safety and Well-being Survey

	Before the pandemic: Not worried n	Before: Not worried % [95% CI]	Before: Somewhat worried n	Before: Somewhat worried % [95% CI]	Before: Very worried n	Before: Very worried % [95% CI]	During the pandemic: Not worried n	During: Not worried % [95% CI]	During: Somewhat worried n	During: Somewhat worried % [95% CI]	During: Very worried n	During: Very worried % [95% CI]	Total n	Total %	During - Before: Not PD	During - Before: Somewhat PD	During - Before: Very PD
Total	1617	50 [47.3-52.8]	1042	39.6 [36.9-42.4]	188	9.2 [7.6-11.2]	1474	46.4 [43.7-49.2]	1131	40.9 [38.2-43.7]	237	11.3 [9.5-13.4]	2,870	100	-3.6[b]	1.3	2.1
Education																	
Less than high school	44	28.4 [21.0-37.1]	71	46.4 [37.7-55.3]	38	21.8 [15.3-30.1]	53	32.3 [24.7-41.1]	60	39 [30.8-48.0]	39	24.8 [17.8-33.4]	158	100	4.0	-7.4	3.0
High school	158	45.4 [38.7-52.3]	164	43.3 [36.7-50.2]	39	10.4 [6.9-15.5]	138	40.8 [34.2-47.7]	165	42.5 [35.9-49.3]	57	15.8 [11.3-21.6]	364	100	-4.6	-0.8	5.4
Some college	511	52.8 [48.3-57.2]	353	39.9 [35.5-44.4]	67	6.7 [4.9-9.0]	473	49.5 [45.0-53.9]	385	41.8 [37.4-46.3]	72	7.9 [5.8-10.8]	938	100	-3.3	1.9	1.3
Bachelor's degree	486	58.8 [53.1-64.2]	253	34.9 [29.8-40.3]	32	5.7 [3.2-10.1]	427	51.4 [45.9-56.9]	297	41 [35.6-46.6]	45	6.2 [3.9-9.7]	775	100	-7.4[b]	6.1	0.5
Advanced degree	418	63.4 [56.9-69.5]	201	31.9 [26.2-38.3]	12	4 [1.7-8.8]	383	57 [50.4-63.4]	224	38.2 [32.0-44.8]	24	4.1 [2.1-7.7]	635	100	-6.4	6.3	0.1
Gender																	
Male	891	51.8 [47.7-55.9]	515	38.3 [34.3-42.4]	87	8.8 [6.5-11.9]	820	48.9 [44.9-53.0]	550	38.1 [34.2-42.1]	119	11.4 [8.8-14.6]	1,506	100	-2.8	-0.2	2.5
Female	726	48.5 [44.7-52.3]	527	40.8 [37.1-44.6]	101	9.6 [7.4-12.3]	654	44.2 [40.5-48.0]	581	43.5 [39.7-47.3]	118	11.3 [8.9-14.1]	1,364	100	-4.3	2.6	1.7
Race/ethnicity																	
White, Non-Latinx	1084	64 [60.2-67.6]	480	31.9 [28.5-35.5]	42	2.9 [1.8-4.5]	975	57.3 [53.5-61.0]	566	36.9 [33.3-40.6]	64	4.5 [3.1-6.4]	1,615	100	-6.7[a]	5.0[b]	1.6
Black, Non-Latinx	62	38.4 [27.9-50.1]	57	54.1 [42.2-65.6]	7	7.3 [2.8-17.7]	67	45.5 [34.1-57.4]	52	46.1 [34.4-58.2]	7	8.2 [3.0-20.4]	127	100	7.1	-8.0	1.0
Asian, Non-Latinx	103	48.9 [40.5-57.4]	90	41.4 [33.3-50.0]	15	9.7 [5.5-16.6]	91	41.1 [33.0-49.6]	100	51 [42.5-59.4]	16	7.1 [4.0-12.5]	208	100	-7.9	9.5	-2.5
Other & 2+ races, Non-Latinx	48	63.6 [45.6-78.4]	21	35.3 [20.6-53.5]	1	0.8 [0.1-5.6]	41	59.9 [42.4-75.1]	28	39.2 [24.0-56.8]	2	1 [0.2-5.0]	71	100	-3.7	3.9	0.2
Latinx	320	34.3 [29.9-39.1]	394	46.1 [41.2-51.1]	123	17.9 [14.2-22.2]	300	34.5 [29.9-39.3]	385	40.8 [36.1-45.7]	148	22.8 [18.6-27.6]	849	100	0.1	-5.3	5.0
Income																	
Less than $25,000	168	33.4 [27.2-40.3]	188	44.5 [37.5-51.6]	67	18.8 [13.5-25.5]	166	36.2 [29.6-43.4]	180	39 [32.4-46.0]	76	21.2 [15.6-28.1]	431	100	2.8	-5.5	2.4
$25,000-$59,999	359	39.8 [34.8-45.1]	309	43.8 [38.5-49.3]	69	15 [11.2-20.0]	323	37.8 [32.8-43.1]	339	44.5 [39.2-49.9]	74	16.3 [12.2-21.4]	744	100	-2.0	0.7	1.2
$60,000-$99,999	450	51.6 [46.0-57.2]	277	43.8 [38.2-49.5]	28	4.5 [2.8-7.3]	417	48.3 [42.8-53.9]	285	42.1 [36.6-47.8]	51	8.9 [6.0-12.8]	757	100	-3.3	-1.7	4.3[a]
$100,000 or more	640	62 [57.1-66.6]	268	32.6 [28.2-37.3]	24	4.6 [2.7-7.8]	568	54.7 [49.9-59.4]	327	38.6 [34.1-43.4]	36	5.8 [3.7-9.0]	938	100	-7.2[a]	6.0[b]	1.2
Age																	

© 2021 Kravitz-Wirtz N et al. *JAMA Network Open.*

18-29	89	42.9 [34.9-51.4]	89	47.2 [38.9-55.7]	20	9 [5.4-14.7]	87	43.6 [35.5-52.0]	91	46.5 [38.2-55.0]	19	8.4 [4.8-14.3]	200	100	0.6	-0.7	-0.6
30-44	212	44.1 [38.4-50.0]	228	40.6 [35.1-46.3]	63	13.9 [10.1-18.8]	200	40.2 [34.6-46.0]	223	40.5 [35.0-46.3]	81	18 [13.7-23.2]	511	100	-4.0	-0.1	4.1
45-59	400	49.4 [44.3-54.4]	279	41.5 [36.5-46.7]	54	8.1 [5.6-11.6]	347	44 [39.0-49.0]	315	44.1 [39.0-49.2]	72	11.1 [8.3-14.7]	738	100	-5.4	2.5	3.0
60+	916	61.3 [57.5-65.0]	446	32.1 [28.7-35.7]	51	5.4 [3.6-8.0]	840	57.4 [53.6-61.1]	502	34.9 [31.5-38.4]	65	6 [4.0-8.9]	1,421	100	-4.0	2.7	0.6
Household gun ownership																	
No guns in home	1058	47.1 [43.8-50.4]	764	41.3 [38.0-44.6]	154	10.6 [8.5-13.0]	979	44.4 [41.1-47.7]	799	41.1 [37.9-44.4]	194	13.2 [10.9-15.8]	1,989	100	-2.8	-0.2	2.6
Gun owner	371	65.8 [58.8-72.1]	147	31.4 [25.2-38.2]	8	2.6 [1.0-6.7]	325	56.6 [49.7-63.3]	185	37.7 [31.4-44.5]	15	5.4 [2.5-11.6]	529	100	-9.2[b]	6.4	2.9
Non-owner living with owner	126	54.1 [44.2-63.6]	81	36.4 [27.6-46.2]	12	9.5 [4.5-19.0]	116	54.2 [44.4-63.6]	90	38.9 [30.0-48.6]	13	6.9 [3.0-15.3]	219	100	0.1	2.5	-2.6
Acquired firearms due to the coronavirus pandemic																	
Non-gun owner	1246	47.2 [44.2-50.3]	895	41.1 [38.1-44.1]	180	10.4 [8.6-12.6]	1149	44.6 [41.6-47.7]	946	41.4 [38.5-44.5]	222	12.4 [10.4-14.7]	2,341	100	-2.6	0.4	1.9
Gun owner, no purchase	365	65.8 [58.8-72.3]	143	31.2 [25.0-38.2]	8	2.6 [1.0-6.9]	321	56.6 [49.6-63.4]	179	37.6 [31.1-44.5]	15	5.6 [2.5-11.9]	519	100	-9.2[b]	6.4	2.9
Gun owner, purchase	6	62 [26.5-88.1]	4	38 [11.9-73.5]	0	0 [NA]	4	54.7 [21.5-84.2]	6	45.3 [15.8-78.5]	0	0 [NA]	10	100	-7.3	7.3	0.0

See footnotes at the end of eAppendix 18.

© 2021 Kravitz-Wirtz N et al. *JAMA Network Open.*

eAppendix 18. Percentage of Respondents Worried About a Violent Event Happening to Them Somewhere Else Before and During the COVID-19 Pandemic, 2020 California Safety and Well-being Survey

	Before the pandemic						During the pandemic						Total		During - Before		
	Not worried		Somewhat worried		Very worried		Not worried		Somewhat worried		Very worried				Not	Somewhat	Very
	n	% [95% CI]	n	% [95% CI]	n	% [95% CI]	n	% [95% CI]	n	% [95% CI]	n	% [95% CI]	n	%	PD	PD	PD
Total	890	27.1 [24.8-29.7]	1531	51.7 [48.9-54.4]	419	19.7 [17.4-22.2]	883	26.7 [24.3-29.1]	1434	48.3 [45.5-51.0]	522	23.5 [21.1-26.0]	2,870	100	-0.5	-3.4[b]	3.8[a]
Education																	
Less than high school	29	19.2 [12.9-27.4]	64	41.9 [33.5-50.9]	60	35.2 [27.2-44.0]	32	20.8 [14.3-29.1]	56	35.9 [27.9-44.8]	62	38.8 [30.6-47.8]	158	100	1.6	-6.0	3.6
High school	102	29.2 [23.3-35.9]	173	46.9 [40.2-53.8]	82	22.4 [17.1-28.8]	92	24.6 [19.2-31.0]	157	45.4 [38.7-52.3]	111	29 [23.2-35.6]	364	100	-4.6	-1.6	6.6
Some college	283	27.1 [23.4-31.2]	520	55.2 [50.7-59.6]	127	16.8 [13.5-20.7]	267	26.1 [22.4-30.1]	503	52.1 [47.6-56.5]	160	20.9 [17.2-25.0]	938	100	-1.0	-3.1	4.0
Bachelor's degree	268	28.7 [24.2-33.8]	415	56.5 [50.8-61.9]	89	13.6 [9.7-18.8]	264	26.9 [22.6-31.7]	404	56.3 [50.7-61.7]	104	15.7 [11.8-20.6]	775	100	-1.9	-0.2	2.1
Advanced degree	208	31 [25.5-37.2]	359	55 [48.5-61.3]	61	12.8 [8.5-18.6]	228	37.2 [31.2-43.7]	314	47.2 [40.8-53.7]	85	14.3 [10.3-19.6]	635	100	6.2	-7.8[b]	1.6
Gender																	
Male	507	29.7 [26.2-33.5]	796	52.8 [48.7-56.8]	185	15.9 [12.9-19.4]	498	28.1 [24.7-31.8]	751	48.6 [44.5-52.7]	240	21.5 [18.1-25.4]	1,506	100	-1.6	-4.2	5.6[a]
Female	383	24.8 [21.7-28.2]	735	50.6 [46.8-54.5]	234	23.1 [19.8-26.7]	385	25.3 [22.2-28.7]	683	48 [44.2-51.8]	282	25.3 [22.0-28.8]	1,364	100	0.5	-2.7	2.2
Race/ethnicity																	
White, Non-Latinx	592	34.7 [31.2-38.3]	885	54.5 [50.7-58.2]	129	9.6 [7.4-12.4]	586	33.4 [30.1-37.0]	841	52.5 [48.7-56.2]	177	12.7 [10.3-15.6]	1,615	100	-1.3	-2.0	3.1[b]
Black, Non-Latinx	40	24.6 [16.1-35.6]	56	43.8 [32.4-55.9]	29	30.5 [20.0-43.4]	44	31.2 [21.3-43.2]	62	47.7 [36.0-59.7]	19	19.9 [11.3-32.7]	127	100	6.6	3.9	-10.6
Asian, Non-Latinx	51	24.1 [17.6-32.1]	118	53.2 [44.6-61.6]	38	21.8 [15.3-30.2]	47	22.6 [16.3-30.5]	109	51.9 [43.4-60.3]	51	24.6 [18.1-32.6]	208	100	-1.5	-1.3	2.8
Other & 2+ races, Non-Latinx	27	34.6 [20.6-52.0]	38	56.9 [39.7-72.5]	5	8.2 [2.7-22.4]	28	34.5 [20.5-51.9]	37	57.1 [40.0-72.7]	6	8.3 [2.8-22.4]	71	100	-0.1	0.3	0.2
Latinx	180	19 [15.4-23.3]	434	48.5 [43.5-53.4]	218	30.2 [25.7-35.0]	178	18.7 [15.1-22.9]	385	40.9 [36.2-45.8]	269	38 [33.2-43.1]	849	100	-0.4	-7.6[a]	7.9[a]
Income																	
Less than $25,000	111	22.8 [17.4-29.3]	209	47.2 [40.2-54.4]	102	26.4 [20.6-33.3]	115	26.3 [20.3-33.3]	191	37.4 [31.0-44.2]	117	32.6 [26.0-39.8]	431	100	3.5	-9.8[a]	6.1
$25,000-$59,999	194	18.1 [14.8-22.0]	409	52.7 [47.2-58.1]	133	27.6 [22.6-33.3]	172	17 [13.6-21.0]	405	49.6 [44.1-55.0]	156	31.5 [26.4-37.2]	744	100	-1.1	-3.2	3.9
$60,000-$99,999	261	30.5 [25.5-35.9]	396	51.8 [46.2-57.4]	96	16.6 [12.6-21.6]	264	28.1 [23.5-33.2]	353	49.7 [44.1-55.3]	134	21.1 [16.8-26.1]	757	100	-2.4	-2.1	4.5
$100,000 or more	324	32.5 [28.3-37.0]	517	52.6 [47.9-57.3]	88	13.8 [10.5-18.0]	332	32 [27.8-36.4]	485	50.9 [46.1-55.6]	115	16.3 [12.8-20.5]	938	100	-0.5	-1.8	2.5

© 2021 Kravitz-Wirtz N et al. *JAMA Network Open.*

Age																	
18-29	34	16.6 [11.1-24.0]	111	55 [46.5-63.2]	51	26.4 [19.6-34.5]	42	18.3 [12.8-25.5]	102	52.6 [44.2-60.9]	52	26.9 [20.0-35.1]	200	100	1.7	-2.4	0.5
30-44	122	26.4 [21.5-32.0]	279	49.9 [44.1-55.7]	101	22.2 [17.6-27.6]	134	27.3 [22.3-32.9]	243	44.6 [38.9-50.4]	125	26.5 [21.6-32.1]	511	100	0.9	-5.3	4.3
45-59	216	27.6 [23.3-32.4]	393	51.5 [46.4-56.6]	122	19.7 [15.7-24.3]	192	24.3 [20.3-28.9]	376	48.2 [43.2-53.3]	163	26 [21.6-30.9]	738	100	-3.3	-3.2	6.3[b]
60+	518	33.8 [30.5-37.2]	748	51.7 [48.0-55.4]	145	12.9 [10.2-16.1]	515	33.2 [30.0-36.7]	713	49.7 [46.0-53.4]	182	15.6 [12.8-19.0]	1,421	100	-0.5	-2.1	2.8
Household gun ownership																	
No guns in home	576	24.9 [22.2-27.9]	1064	51.5 [48.1-54.8]	330	22.2 [19.4-25.3]	594	26.2 [23.4-29.2]	980	46.6 [43.3-49.9]	394	25.5 [22.6-28.7]	1,989	100	1.3	-4.9[a]	3.3
Gun owner	215	38.5 [32.1-45.2]	282	54.2 [47.4-60.8]	30	6.9 [4.4-10.6]	193	29.1 [23.9-35.0]	272	53.3 [46.5-59.9]	62	17.2 [12.2-23.9]	529	100	-9.4[a]	-0.9	10.4[a]
Non-owner living with owner	61	27.9 [19.9-37.7]	122	49.8 [40.2-59.5]	36	22.2 [14.6-32.3]	65	30.2 [21.9-40.0]	114	48.2 [38.6-57.9]	40	21.6 [14.4-31.3]	219	100	2.3	-1.7	-0.6
Acquired firearms due to the coronavirus pandemic																	
Non-gun owner	675	25.1 [22.6-27.8]	1249	51.2 [48.1-54.3]	389	21.9 [19.3-24.8]	690	26.2 [23.7-29.0]	1162	47.4 [44.3-50.4]	460	24.6 [21.9-27.4]	2,341	100	1.1	-3.8[b]	2.6
Gun owner, no purchase	209	37.5 [31.1-44.3]	279	55.1 [48.3-61.8]	29	6.9 [4.4-10.8]	189	28.2 [22.9-34.0]	268	54.1 [47.2-60.8]	60	17.4 [12.2-24.2]	519	100	-9.3[a]	-1.1	10.5[a]
Gun owner, purchase	6	79.7 [50.1-93.9]	3	15.4 [4.0-44.0]	1	5 [0.6-30.2]	4	68.5 [35.9-89.4]	4	22 [6.2-54.8]	2	9.5 [2.0-34.4]	10	100	-11.1	6.6	4.5

PD = Prevalence difference; CI = Confidence interval

Rows and columns may not sum to total because of missing values.

Counts are unweighted; percentages and 95% CIs are survey-weighted.

To ascertain race-ethnicity, respondents were asked, "Are you Spanish, Hispanic, or Latino?" and then, "Please indicate what you consider your race to be. We appreciate your effort to describe your background using these US Census Bureau categories." Response options were White, Black or African American, American Indian or Alaska Native, Asian, Native Hawaiian or other Pacific Islander, and Some other race. Respondents could select all answers that apply. American Indian or Alaska Native, Native Hawaiian or other Pacific Islander, Some other race, and those who selected more than one race were combined by Ipsos into an Other & 2+ races category.

[a]$P<0.05$

[b]$P<0.10$

© 2021 Kravitz-Wirtz N et al. *JAMA Network Open.*
